# Improvement in accuracy of multiple sequence alignment using novel group-to-group sequence alignment algorithm with piecewise linear gap cost

**DOI:** 10.1186/1471-2105-7-524

**Published:** 2006-12-01

**Authors:** Shinsuke Yamada, Osamu Gotoh, Hayato Yamana

**Affiliations:** 1 Department of Computer Science, Graduate School of Science and Engineering, Waseda University, 3-4-1 Okubo, Shinjuku-ku, Tokyo 169-8555, Japan; 2 Computational Biology Research Center (CBRC), National Institute of Advanced Industrial Science and Technology (AIST), 2-43 Aomi, Koto-ku, Tokyo 135-0064, Japan; 3 Department of Intelligence Science and Technology, Graduate School of Informatics, Kyoto University, Yoshida-Honmachi, Sakyo-ku, Kyoto 606-8501, Japan

## Abstract

**Background:**

Multiple sequence alignment (MSA) is a useful tool in bioinformatics. Although many MSA algorithms have been developed, there is still room for improvement in accuracy and speed. In the alignment of a family of protein sequences, global MSA algorithms perform better than local ones in many cases, while local ones perform better than global ones when some sequences have long insertions or deletions (indels) relative to others. Many recent leading MSA algorithms have incorporated pairwise alignment information obtained from a mixture of sources into their scoring system to improve accuracy of alignment containing long indels.

**Results:**

We propose a novel group-to-group sequence alignment algorithm that uses a piecewise linear gap cost. We developed a program called PRIME, which employs our proposed algorithm to optimize the well-defined sum-of-pairs score. PRIME stands for Profile-based Randomized Iteration MEthod. We evaluated PRIME and some recent MSA programs using BAliBASE version 3.0 and PREFAB version 4.0 benchmarks. The results of benchmark tests showed that PRIME can construct accurate alignments comparable to the most accurate programs currently available, including L-INS-i of MAFFT, ProbCons, and T-Coffee.

**Conclusion:**

PRIME enables users to construct accurate alignments without having to employ pairwise alignment information. PRIME is available at .

## Background

Multiple sequence alignment (MSA) is a useful tool for elucidating the relationships among function, evolution, sequence, and structure of biological macromolecules such as genes and proteins [1-3]. Although we can calculate the optimal alignment of a set of sequences by *n*-dimensional dynamic programming (DP), the DP method is applicable to only a small number of sequences. In fact, even when a sum-of-pairs (SP) score with the simplest gap cost is used as an objective function, computation of optimal MSA is an NP-hard problem [4]. Hence, many heuristic methods have been developed. Almost all practical methods presently available adopt either a progressive [5-7] or an iterative [8-10] heuristic strategy.

The group-to-group sequence alignment algorithm is a straightforward extension of the pairwise sequence alignment algorithm, and is the core of progressive and iterative methods. The essential difference of group-to-group sequence alignment from pairwise sequence alignment is the existence of gaps within each group of prealigned sequences. The gap opening penalty used in an affine gap cost disrupts the independence between adjacent columns, and hence calculating the optimal alignment between two groups with respect to the SP score was shown to be NP-complete [11]. Gotoh was the first to devise a group-to-group sequence alignment algorithm that optimizes the SP score by using a candidate list paradigm [12]. An algorithm with a candidate list paradigm, similar to the branch-and-bound method, prunes the candidates that are dispensable for arrival at an optimal solution. Kececioglu and Starrett proposed another candidate-pruning method [11]. Although these algorithms can calculate the optimal alignment between two groups, they require relatively extensive computational resources. Several papers have reported faster algorithms that use the heuristic estimation of gap opening penalties [8,10].

Several studies have discussed the tendency that global alignment methods perform better than local ones [13,14]. However, the opposite is also true when some sequences to be aligned have long insertions or deletions (indels). One reason for this tendency is that almost all group-to-group sequence alignment algorithms use an affine-like gap cost that over-penalizes long indels. To alleviate this problem, several methods have combined pairwise global and local alignments, or incorporated consistency information among pairwise alignments [6,15,16]. Another strategy to prevent over-penalizing long indels is to use a concave function as the gap cost. It is relatively easy to choose a concave gap cost that does not over-penalize long indels, and several pairwise sequence alignment algorithms using this gap cost have been developed [17,18]. However, there have been few attempts to incorporate this gap cost into a group-to-group sequence alignment algorithm to develop an MSA program.

In this paper, we propose a novel group-to-group sequence alignment algorithm with a piecewise linear gap cost [18], which is the key to a progressive or an iterative refinement method. The piecewise linear gap cost [18] is one of the concave functions and consists of *L *linear functions. Depending on the gap length, this gap cost varies its inclination, which corresponds to the gap extension penalty. However, in the case of group-to-group sequence alignment algorithm, it is difficult to calculate the proper gap extension penalty with only the data structures used in the previous algorithm that were designed to detect the opening of new gaps [12,19]. Accordingly, we newly introduce two additional data structures: 'insertion length profile' and 'dynamic gap information'. An insertion length profile vector is associated with each column of a group of sequences, while dynamic gap information keeps track of information about gaps inserted into a group during the DP process. Together with those used in the previous algorithm, gap extension penalty can be calculated efficiently. Using the proposed algorithm, we developed a program called PRIME.

PRIME stands for Profile-based Randomized Iteration MEthod. As a result of benchmark tests, the accuracy of our method is shown to be comparable to the most accurate methods available today, all of which incorporate pairwise alignment information obtained from all-by-all pairwise alignment. This implies that the piecewise linear gap cost is as effective as pairwise alignment information in improving the alignment accuracy of sequences, some of which have long indels.

## Algorithms

In this section, we first review the previous group-to-group sequence alignment algorithm with an affine gap cost [12,19], and then describe a novel one with a piecewise linear gap cost. The final subsection outlines a doubly nested randomized iterative strategy with which our proposed algorithm is integrated. 

The definitions of symbols are as follows. Let Σ be the residue set and |Σ|, the number of elements in Σ. Σ* denotes the set containing a null and each element in Σ. A null means that a residue of one sequence does not aligned with that of another sequence when aligning sequences, and is denoted by the symbol '-'. *A *and *B *denote prealigned groups of sequences. *A *includes *m *rows, and *B*, *n *rows. The respective lengths of *A *and *B *are *I *and *J*. *A*_*p*_, **a**_*i*_, and *a*_*p*,*i *_denote the *p*-th row of *A*, the *i*-th column of *A *and the *i*-th residue of *A*_*p*_, respectively. *B*_*q*_, **b**_*j*_, and *b*_*q*,*j *_are defined similarly. Both *a*_*p*,*i *_and *b*_*q*,*j *_belong to Σ*. Note that any column of a group must not be a null column, which consists of nulls only. If all nulls are removed from a group, each row is an usual sequence. A run of consecutive nulls in a row is called a gap. A gap length is the number of nulls constituting the gap. A segment of *A *that consists of consecutive columns **a**_*s *_to **a**_*t *_is denoted by *A*(*s*, *t*); *A *is also expressed as *A*(1, *I*). *s*(*a*, *b*) is a substitution score between residues *a *and *b*. By *g*(*x*), we mean a gap cost function of gap length *x*. The pair weight between the *p*-th sequence in *A *and the *q*-th sequence in *B *is *w*_*p*,*q*_. If the three-way method [20] is used to calculate the pair weights, *w*_*p*,*q *_can be factorized as wAp
 MathType@MTEF@5@5@+=feaafiart1ev1aaatCvAUfKttLearuWrP9MDH5MBPbIqV92AaeXatLxBI9gBaebbnrfifHhDYfgasaacH8akY=wiFfYdH8Gipec8Eeeu0xXdbba9frFj0=OqFfea0dXdd9vqai=hGuQ8kuc9pgc9s8qqaq=dirpe0xb9q8qiLsFr0=vr0=vr0dc8meaabaqaciaacaGaaeqabaqabeGadaaakeaacqWG3bWDdaWgaaWcbaGaemyqae0aaSbaaWqaaiabdchaWbqabaaaleqaaaaa@30FB@·wBq
 MathType@MTEF@5@5@+=feaafiart1ev1aaatCvAUfKttLearuWrP9MDH5MBPbIqV92AaeXatLxBI9gBaebbnrfifHhDYfgasaacH8akY=wiFfYdH8Gipec8Eeeu0xXdbba9frFj0=OqFfea0dXdd9vqai=hGuQ8kuc9pgc9s8qqaq=dirpe0xb9q8qiLsFr0=vr0=vr0dc8meaabaqaciaacaGaaeqabaqabeGadaaakeaacqWG3bWDdaWgaaWcbaGaemOqai0aaSbaaWqaaiabdghaXbqabaaaleqaaaaa@30FF@, where wAp
 MathType@MTEF@5@5@+=feaafiart1ev1aaatCvAUfKttLearuWrP9MDH5MBPbIqV92AaeXatLxBI9gBaebbnrfifHhDYfgasaacH8akY=wiFfYdH8Gipec8Eeeu0xXdbba9frFj0=OqFfea0dXdd9vqai=hGuQ8kuc9pgc9s8qqaq=dirpe0xb9q8qiLsFr0=vr0=vr0dc8meaabaqaciaacaGaaeqabaqabeGadaaakeaacqWG3bWDdaWgaaWcbaGaemyqae0aaSbaaWqaaiabdchaWbqabaaaleqaaaaa@30FB@ and wBq
 MathType@MTEF@5@5@+=feaafiart1ev1aaatCvAUfKttLearuWrP9MDH5MBPbIqV92AaeXatLxBI9gBaebbnrfifHhDYfgasaacH8akY=wiFfYdH8Gipec8Eeeu0xXdbba9frFj0=OqFfea0dXdd9vqai=hGuQ8kuc9pgc9s8qqaq=dirpe0xb9q8qiLsFr0=vr0=vr0dc8meaabaqaciaacaGaaeqabaqabeGadaaakeaacqWG3bWDdaWgaaWcbaGaemOqai0aaSbaaWqaaiabdghaXbqabaaaleqaaaaa@30FF@ are the weights for the *p*-th sequence in *A *and the *q*-th one in *B*, respectively.

### Review of previous group-to-group sequence alignment algorithm with affine gap cost

The previous group-to-group sequence alignment algorithm that optimizes SP or weighted SP score with an affine gap cost is based on a two-dimensional DP method [12]. The key point of this algorithm is to exactly evaluate the gap opening penalties during the DP process. To explicitly consider gaps already present in each group, this algorithm introduces a gap state that denotes the number of consecutive nulls up to the current position.

Another important feature of this algorithm is the candidate list paradigm, which is a variant of branch-and-bound methods. Because the calculation of gap opening penalties depends on a previous partial DP path, simple extension of pairwise sequence alignment algorithm may not yield globally optimal alignment between two groups [12]. For rigorous calculation, not only locally optimal partial paths but also those that possibly contribute to globally optimal alignment have to be stored at each node of a DP matrix [11,12]. In the worst case, the number of candidates to be stored grows exponentially with the total number of sequences in the two groups [11]. As discussed in some papers [8,10,12], the group-to-group sequence alignment algorithm without the candidate list paradigm may suffice for good alignment. Moreover, because the novel group-to-group sequence alignment algorithm described below requires roughly twice as much computation time as the previous one at each DP process, we adopted a simpler algorithm without the candidate list paradigm.

#### Basic algorithm

Let an affine gap cost function be *g*(*x*) = -(*ux *+ *v*), where *u*(> 0) and *v*(> 0) are constants called gap extension penalty and gap opening penalty, respectively. The group-to-group sequence alignment algorithm with the affine gap cost employs essentially the same recurrent relations as the pairwise sequence alignment algorithm [21], with exact evaluation of gap opening and extension penalties. Like the pairwise sequence alignment algorithm, we calculate four variables at each node, (*i*, *j*), of the DP matrix: Hi,j0
 MathType@MTEF@5@5@+=feaafiart1ev1aaatCvAUfKttLearuWrP9MDH5MBPbIqV92AaeXatLxBI9gBaebbnrfifHhDYfgasaacH8akY=wiFfYdH8Gipec8Eeeu0xXdbba9frFj0=OqFfea0dXdd9vqai=hGuQ8kuc9pgc9s8qqaq=dirpe0xb9q8qiLsFr0=vr0=vr0dc8meaabaqaciaacaGaaeqabaqabeGadaaakeaacqWGibasdaqhaaWcbaGaemyAaKMaeiilaWIaemOAaOgabaGaeGimaadaaaaa@3278@, Hi,j1
 MathType@MTEF@5@5@+=feaafiart1ev1aaatCvAUfKttLearuWrP9MDH5MBPbIqV92AaeXatLxBI9gBaebbnrfifHhDYfgasaacH8akY=wiFfYdH8Gipec8Eeeu0xXdbba9frFj0=OqFfea0dXdd9vqai=hGuQ8kuc9pgc9s8qqaq=dirpe0xb9q8qiLsFr0=vr0=vr0dc8meaabaqaciaacaGaaeqabaqabeGadaaakeaacqWGibasdaqhaaWcbaGaemyAaKMaeiilaWIaemOAaOgabaGaeGymaedaaaaa@327A@, Hi,j2
 MathType@MTEF@5@5@+=feaafiart1ev1aaatCvAUfKttLearuWrP9MDH5MBPbIqV92AaeXatLxBI9gBaebbnrfifHhDYfgasaacH8akY=wiFfYdH8Gipec8Eeeu0xXdbba9frFj0=OqFfea0dXdd9vqai=hGuQ8kuc9pgc9s8qqaq=dirpe0xb9q8qiLsFr0=vr0=vr0dc8meaabaqaciaacaGaaeqabaqabeGadaaakeaacqWGibasdaqhaaWcbaGaemyAaKMaeiilaWIaemOAaOgabaGaeGOmaidaaaaa@327C@, and Hi,j3
 MathType@MTEF@5@5@+=feaafiart1ev1aaatCvAUfKttLearuWrP9MDH5MBPbIqV92AaeXatLxBI9gBaebbnrfifHhDYfgasaacH8akY=wiFfYdH8Gipec8Eeeu0xXdbba9frFj0=OqFfea0dXdd9vqai=hGuQ8kuc9pgc9s8qqaq=dirpe0xb9q8qiLsFr0=vr0=vr0dc8meaabaqaciaacaGaaeqabaqabeGadaaakeaacqWGibasdaqhaaWcbaGaemyAaKMaeiilaWIaemOAaOgabaGaeG4mamdaaaaa@327E@. Hi,j0
 MathType@MTEF@5@5@+=feaafiart1ev1aaatCvAUfKttLearuWrP9MDH5MBPbIqV92AaeXatLxBI9gBaebbnrfifHhDYfgasaacH8akY=wiFfYdH8Gipec8Eeeu0xXdbba9frFj0=OqFfea0dXdd9vqai=hGuQ8kuc9pgc9s8qqaq=dirpe0xb9q8qiLsFr0=vr0=vr0dc8meaabaqaciaacaGaaeqabaqabeGadaaakeaacqWGibasdaqhaaWcbaGaemyAaKMaeiilaWIaemOAaOgabaGaeGimaadaaaaa@3278@ holds the best score among Hi,j1
 MathType@MTEF@5@5@+=feaafiart1ev1aaatCvAUfKttLearuWrP9MDH5MBPbIqV92AaeXatLxBI9gBaebbnrfifHhDYfgasaacH8akY=wiFfYdH8Gipec8Eeeu0xXdbba9frFj0=OqFfea0dXdd9vqai=hGuQ8kuc9pgc9s8qqaq=dirpe0xb9q8qiLsFr0=vr0=vr0dc8meaabaqaciaacaGaaeqabaqabeGadaaakeaacqWGibasdaqhaaWcbaGaemyAaKMaeiilaWIaemOAaOgabaGaeGymaedaaaaa@327A@, Hi,j2
 MathType@MTEF@5@5@+=feaafiart1ev1aaatCvAUfKttLearuWrP9MDH5MBPbIqV92AaeXatLxBI9gBaebbnrfifHhDYfgasaacH8akY=wiFfYdH8Gipec8Eeeu0xXdbba9frFj0=OqFfea0dXdd9vqai=hGuQ8kuc9pgc9s8qqaq=dirpe0xb9q8qiLsFr0=vr0=vr0dc8meaabaqaciaacaGaaeqabaqabeGadaaakeaacqWGibasdaqhaaWcbaGaemyAaKMaeiilaWIaemOAaOgabaGaeGOmaidaaaaa@327C@, and Hi,j3
 MathType@MTEF@5@5@+=feaafiart1ev1aaatCvAUfKttLearuWrP9MDH5MBPbIqV92AaeXatLxBI9gBaebbnrfifHhDYfgasaacH8akY=wiFfYdH8Gipec8Eeeu0xXdbba9frFj0=OqFfea0dXdd9vqai=hGuQ8kuc9pgc9s8qqaq=dirpe0xb9q8qiLsFr0=vr0=vr0dc8meaabaqaciaacaGaaeqabaqabeGadaaakeaacqWGibasdaqhaaWcbaGaemyAaKMaeiilaWIaemOAaOgabaGaeG4mamdaaaaa@327E@ at (*i*, *j*). Hi,j1
 MathType@MTEF@5@5@+=feaafiart1ev1aaatCvAUfKttLearuWrP9MDH5MBPbIqV92AaeXatLxBI9gBaebbnrfifHhDYfgasaacH8akY=wiFfYdH8Gipec8Eeeu0xXdbba9frFj0=OqFfea0dXdd9vqai=hGuQ8kuc9pgc9s8qqaq=dirpe0xb9q8qiLsFr0=vr0=vr0dc8meaabaqaciaacaGaaeqabaqabeGadaaakeaacqWGibasdaqhaaWcbaGaemyAaKMaeiilaWIaemOAaOgabaGaeGymaedaaaaa@327A@ is a score of a partial alignment where **a**_*i *_and **b**_*j *_are aligned. Hi,j2
 MathType@MTEF@5@5@+=feaafiart1ev1aaatCvAUfKttLearuWrP9MDH5MBPbIqV92AaeXatLxBI9gBaebbnrfifHhDYfgasaacH8akY=wiFfYdH8Gipec8Eeeu0xXdbba9frFj0=OqFfea0dXdd9vqai=hGuQ8kuc9pgc9s8qqaq=dirpe0xb9q8qiLsFr0=vr0=vr0dc8meaabaqaciaacaGaaeqabaqabeGadaaakeaacqWGibasdaqhaaWcbaGaemyAaKMaeiilaWIaemOAaOgabaGaeGOmaidaaaaa@327C@ and Hi,j3
 MathType@MTEF@5@5@+=feaafiart1ev1aaatCvAUfKttLearuWrP9MDH5MBPbIqV92AaeXatLxBI9gBaebbnrfifHhDYfgasaacH8akY=wiFfYdH8Gipec8Eeeu0xXdbba9frFj0=OqFfea0dXdd9vqai=hGuQ8kuc9pgc9s8qqaq=dirpe0xb9q8qiLsFr0=vr0=vr0dc8meaabaqaciaacaGaaeqabaqabeGadaaakeaacqWGibasdaqhaaWcbaGaemyAaKMaeiilaWIaemOAaOgabaGaeG4mamdaaaaa@327E@ mean partial alignment scores where **a**_*i *_and **b**_*j *_are aligned with null columns, respectively. The recurrent equations are:

Hi,j0=max⁡1≤k≤3{Hi,jk}     (1)
 MathType@MTEF@5@5@+=feaafiart1ev1aaatCvAUfKttLearuWrP9MDH5MBPbIqV92AaeXatLxBI9gBaebbnrfifHhDYfgasaacH8akY=wiFfYdH8Gipec8Eeeu0xXdbba9frFj0=OqFfea0dXdd9vqai=hGuQ8kuc9pgc9s8qqaq=dirpe0xb9q8qiLsFr0=vr0=vr0dc8meaabaqaciaacaGaaeqabaqabeGadaaakeaacqWGibasdaqhaaWcbaGaemyAaKMaeiilaWIaemOAaOgabaGaeGimaadaaOGaeyypa0ZaaCbeaeaacyGGTbqBcqGGHbqycqGG4baEaSqaaiabigdaXiabgsMiJkabdUgaRjabgsMiJkabiodaZaqabaGcdaGadeqaaiabdIeainaaDaaaleaacqWGPbqAcqGGSaalcqWGQbGAaeaacqWGRbWAaaaakiaawUhacaGL9baacaWLjaGaaCzcamaabmaabaGaeGymaedacaGLOaGaayzkaaaaaa@4AD4@

Hi,j1=Hi−1,j−10+G(ai,bj;Pi−1,j−10)+S(ai,bj)     (2)
 MathType@MTEF@5@5@+=feaafiart1ev1aaatCvAUfKttLearuWrP9MDH5MBPbIqV92AaeXatLxBI9gBaebbnrfifHhDYfgasaacH8akY=wiFfYdH8Gipec8Eeeu0xXdbba9frFj0=OqFfea0dXdd9vqai=hGuQ8kuc9pgc9s8qqaq=dirpe0xb9q8qiLsFr0=vr0=vr0dc8meaabaqaciaacaGaaeqabaqabeGadaaakeaacqWGibasdaqhaaWcbaGaemyAaKMaeiilaWIaemOAaOgabaGaeGymaedaaOGaeyypa0JaemisaG0aa0baaSqaaiabdMgaPjabgkHiTiabigdaXiabcYcaSiabdQgaQjabgkHiTiabigdaXaqaaiabicdaWaaakiabgUcaRiabdEeahjabcIcaOGqabiab=fgaHnaaBaaaleaacqWGPbqAaeqaaOGaeiilaWIae8Nyai2aaSbaaSqaaiabdQgaQbqabaGccqGG7aWocqWGqbaudaqhaaWcbaGaemyAaKMaeyOeI0IaeGymaeJaeiilaWIaemOAaOMaeyOeI0IaeGymaedabaGaeGimaadaaOGaeiykaKIaey4kaSIaem4uamLaeiikaGIae8xyae2aaSbaaSqaaiabdMgaPbqabaGccqGGSaalcqWFIbGydaWgaaWcbaGaemOAaOgabeaakiabcMcaPiaaxMaacaWLjaWaaeWaaeaacqaIYaGmaiaawIcacaGLPaaaaaa@6017@

Hi,j2=max⁡k=0,2{Hi−1,jk+G(ai,−;Pi−1,jk)}+S(ai,−)     (3)
 MathType@MTEF@5@5@+=feaafiart1ev1aaatCvAUfKttLearuWrP9MDH5MBPbIqV92AaeXatLxBI9gBaebbnrfifHhDYfgasaacH8akY=wiFfYdH8Gipec8Eeeu0xXdbba9frFj0=OqFfea0dXdd9vqai=hGuQ8kuc9pgc9s8qqaq=dirpe0xb9q8qiLsFr0=vr0=vr0dc8meaabaqaciaacaGaaeqabaqabeGadaaakeaacqWGibasdaqhaaWcbaGaemyAaKMaeiilaWIaemOAaOgabaGaeGOmaidaaOGaeyypa0ZaaCbeaeaacyGGTbqBcqGGHbqycqGG4baEaSqaaiabdUgaRjabg2da9iabicdaWiabcYcaSiabikdaYaqabaGcdaGadeqaaiabdIeainaaDaaaleaacqWGPbqAcqGHsislcqaIXaqmcqGGSaalcqWGQbGAaeaacqWGRbWAaaGccqGHRaWkcqWGhbWrcqGGOaakieqacqWFHbqydaWgaaWcbaGaemyAaKgabeaakiabcYcaSiabgkHiTiabcUda7iabdcfaqnaaDaaaleaacqWGPbqAcqGHsislcqaIXaqmcqGGSaalcqWGQbGAaeaacqWGRbWAaaGccqGGPaqkaiaawUhacaGL9baacqGHRaWkcqWGtbWucqGGOaakcqWFHbqydaWgaaWcbaGaemyAaKgabeaakiabcYcaSiabgkHiTiabcMcaPiaaxMaacaWLjaWaaeWaaeaacqaIZaWmaiaawIcacaGLPaaaaaa@6525@

Hi,j3=max⁡k=0,3{Hi,j−1k+G(−,bj;Pi,j−1k)}+S(−,bj)     (4)
 MathType@MTEF@5@5@+=feaafiart1ev1aaatCvAUfKttLearuWrP9MDH5MBPbIqV92AaeXatLxBI9gBaebbnrfifHhDYfgasaacH8akY=wiFfYdH8Gipec8Eeeu0xXdbba9frFj0=OqFfea0dXdd9vqai=hGuQ8kuc9pgc9s8qqaq=dirpe0xb9q8qiLsFr0=vr0=vr0dc8meaabaqaciaacaGaaeqabaqabeGadaaakeaacqWGibasdaqhaaWcbaGaemyAaKMaeiilaWIaemOAaOgabaGaeG4mamdaaOGaeyypa0ZaaCbeaeaacyGGTbqBcqGGHbqycqGG4baEaSqaaiabdUgaRjabg2da9iabicdaWiabcYcaSiabiodaZaqabaGcdaGadeqaaiabdIeainaaDaaaleaacqWGPbqAcqGGSaalcqWGQbGAcqGHsislcqaIXaqmaeaacqWGRbWAaaGccqGHRaWkcqWGhbWrcqGGOaakcqGHsislcqGGSaalieqacqWFIbGydaWgaaWcbaGaemOAaOgabeaakiabcUda7iabdcfaqnaaDaaaleaacqWGPbqAcqGGSaalcqWGQbGAcqGHsislcqaIXaqmaeaacqWGRbWAaaGccqGGPaqkaiaawUhacaGL9baacqGHRaWkcqWGtbWucqGGOaakcqGHsislcqGGSaalcqWFIbGydaWgaaWcbaGaemOAaOgabeaakiabcMcaPiaaxMaacaWLjaWaaeWaaeaacqaI0aanaiaawIcacaGLPaaaaaa@6533@

where '-' denotes a null column, and Pi−1,j−10
 MathType@MTEF@5@5@+=feaafiart1ev1aaatCvAUfKttLearuWrP9MDH5MBPbIqV92AaeXatLxBI9gBaebbnrfifHhDYfgasaacH8akY=wiFfYdH8Gipec8Eeeu0xXdbba9frFj0=OqFfea0dXdd9vqai=hGuQ8kuc9pgc9s8qqaq=dirpe0xb9q8qiLsFr0=vr0=vr0dc8meaabaqaciaacaGaaeqabaqabeGadaaakeaacqWGqbaudaqhaaWcbaGaemyAaKMaeyOeI0IaeGymaeJaeiilaWIaemOAaOMaeyOeI0IaeGymaedabaGaeGimaadaaaaa@3642@ is a partial DP path. A partial DP path is one from (0, 0) to the node of the DP matrix in question, representing a partial alignment; for example, Pi,j0
 MathType@MTEF@5@5@+=feaafiart1ev1aaatCvAUfKttLearuWrP9MDH5MBPbIqV92AaeXatLxBI9gBaebbnrfifHhDYfgasaacH8akY=wiFfYdH8Gipec8Eeeu0xXdbba9frFj0=OqFfea0dXdd9vqai=hGuQ8kuc9pgc9s8qqaq=dirpe0xb9q8qiLsFr0=vr0=vr0dc8meaabaqaciaacaGaaeqabaqabeGadaaakeaacqWGqbaudaqhaaWcbaGaemyAaKMaeiilaWIaemOAaOgabaGaeGimaadaaaaa@3288@ represents a partial alignment with the best score Hi,j0
 MathType@MTEF@5@5@+=feaafiart1ev1aaatCvAUfKttLearuWrP9MDH5MBPbIqV92AaeXatLxBI9gBaebbnrfifHhDYfgasaacH8akY=wiFfYdH8Gipec8Eeeu0xXdbba9frFj0=OqFfea0dXdd9vqai=hGuQ8kuc9pgc9s8qqaq=dirpe0xb9q8qiLsFr0=vr0=vr0dc8meaabaqaciaacaGaaeqabaqabeGadaaakeaacqWGibasdaqhaaWcbaGaemyAaKMaeiilaWIaemOAaOgabaGaeGimaadaaaaa@3278@ between *A*(1, *i*) and *B*(1, *j*)·*S*(**a**_*i*_, **b**_*j*_) is responsible for the calculation of substitution scores and gap extension penalties:

S(ai,bj)=∑1≤p≤m∑1≤q≤nwp,q⋅s(ap,i,bq,j).     (5)
 MathType@MTEF@5@5@+=feaafiart1ev1aaatCvAUfKttLearuWrP9MDH5MBPbIqV92AaeXatLxBI9gBaebbnrfifHhDYfgasaacH8akY=wiFfYdH8Gipec8Eeeu0xXdbba9frFj0=OqFfea0dXdd9vqai=hGuQ8kuc9pgc9s8qqaq=dirpe0xb9q8qiLsFr0=vr0=vr0dc8meaabaqaciaacaGaaeqabaqabeGadaaakeaacqWGtbWucqGGOaakieqacqWFHbqydaWgaaWcbaGaemyAaKgabeaakiabcYcaSiab=jgaInaaBaaaleaacqWGQbGAaeqaaOGaeiykaKIaeyypa0ZaaabuaeaadaaeqbqaaiabdEha3naaBaaaleaacqWGWbaCcqGGSaalcqWGXbqCaeqaaaqaaiabigdaXiabgsMiJkabdghaXjabgsMiJkabd6gaUbqab0GaeyyeIuoakiabgwSixlabdohaZjabcIcaOiabdggaHnaaBaaaleaacqWGWbaCcqGGSaalcqWGPbqAaeqaaOGaeiilaWIaemOyai2aaSbaaSqaaiabdghaXjabcYcaSiabdQgaQbqabaGccqGGPaqkaSqaaiabigdaXiabgsMiJkabdchaWjabgsMiJkabd2gaTbqab0GaeyyeIuoakiabc6caUiaaxMaacaWLjaWaaeWaaeaacqaI1aqnaiaawIcacaGLPaaaaaa@646B@

If either *a *or *b *is a null, *s*(*a*, *b*) = -*u*, and if both *a *and *b *are null, *s*(*a*,* b*) = 0. *G*(**a**_*i*_, **b**_*j*_; Pi−1,j−10
 MathType@MTEF@5@5@+=feaafiart1ev1aaatCvAUfKttLearuWrP9MDH5MBPbIqV92AaeXatLxBI9gBaebbnrfifHhDYfgasaacH8akY=wiFfYdH8Gipec8Eeeu0xXdbba9frFj0=OqFfea0dXdd9vqai=hGuQ8kuc9pgc9s8qqaq=dirpe0xb9q8qiLsFr0=vr0=vr0dc8meaabaqaciaacaGaaeqabaqabeGadaaakeaacqWGqbaudaqhaaWcbaGaemyAaKMaeyOeI0IaeGymaeJaeiilaWIaemOAaOMaeyOeI0IaeGymaedabaGaeGimaadaaaaa@3642@) is the gap opening penalty when **a**_*i *_is aligned with **b**_*j*_:

G(ai,bj;Pi−1,j−10)=∑1≤p≤m∑1≤q≤nwp,q⋅(−v)⋅γ(ap,i,bq,j,xp,i−10,yq,j−10).     (6)
 MathType@MTEF@5@5@+=feaafiart1ev1aaatCvAUfKttLearuWrP9MDH5MBPbIqV92AaeXatLxBI9gBaebbnrfifHhDYfgasaacH8akY=wiFfYdH8Gipec8Eeeu0xXdbba9frFj0=OqFfea0dXdd9vqai=hGuQ8kuc9pgc9s8qqaq=dirpe0xb9q8qiLsFr0=vr0=vr0dc8meaabaqaciaacaGaaeqabaqabeGadaaakeaacqWGhbWrcqGGOaakieqacqWFHbqydaWgaaWcbaGaemyAaKgabeaakiabcYcaSiab=jgaInaaBaaaleaacqWGQbGAaeqaaOGaei4oaSJaemiuaa1aa0baaSqaaiabdMgaPjabgkHiTiabigdaXiabcYcaSiabdQgaQjabgkHiTiabigdaXaqaaiabicdaWaaakiabcMcaPiabg2da9maaqafabaWaaabuaeaacqWG3bWDdaWgaaWcbaGaemiCaaNaeiilaWIaemyCaehabeaaaeaacqaIXaqmcqGHKjYOcqWGXbqCcqGHKjYOcqWGUbGBaeqaniabggHiLdaaleaacqaIXaqmcqGHKjYOcqWGWbaCcqGHKjYOcqWGTbqBaeqaniabggHiLdGccqGHflY1cqGGOaakcqGHsislcqWG2bGDcqGGPaqkcqGHflY1iiGacqGFZoWzcqGGOaakcqWGHbqydaWgaaWcbaGaemiCaaNaeiilaWIaemyAaKgabeaakiabcYcaSiabdkgaInaaBaaaleaacqWGXbqCcqGGSaalcqWGQbGAaeqaaOGaeiilaWIaemiEaG3aa0baaSqaaiabdchaWjabcYcaSiabdMgaPjabgkHiTiabigdaXaqaaiabicdaWaaakiabcYcaSiabdMha5naaDaaaleaacqWGXbqCcqGGSaalcqWGQbGAcqGHsislcqaIXaqmaeaacqaIWaamaaGccqGGPaqkcqGGUaGlcaWLjaGaaCzcamaabmaabaGaeGOnaydacaGLOaGaayzkaaaaaa@8789@

xp,i−10
 MathType@MTEF@5@5@+=feaafiart1ev1aaatCvAUfKttLearuWrP9MDH5MBPbIqV92AaeXatLxBI9gBaebbnrfifHhDYfgasaacH8akY=wiFfYdH8Gipec8Eeeu0xXdbba9frFj0=OqFfea0dXdd9vqai=hGuQ8kuc9pgc9s8qqaq=dirpe0xb9q8qiLsFr0=vr0=vr0dc8meaabaqaciaacaGaaeqabaqabeGadaaakeaacqWG4baEdaqhaaWcbaGaemiCaaNaeiilaWIaemyAaKMaeyOeI0IaeGymaedabaGaeGimaadaaaaa@34C1@ and yq,j−10
 MathType@MTEF@5@5@+=feaafiart1ev1aaatCvAUfKttLearuWrP9MDH5MBPbIqV92AaeXatLxBI9gBaebbnrfifHhDYfgasaacH8akY=wiFfYdH8Gipec8Eeeu0xXdbba9frFj0=OqFfea0dXdd9vqai=hGuQ8kuc9pgc9s8qqaq=dirpe0xb9q8qiLsFr0=vr0=vr0dc8meaabaqaciaacaGaaeqabaqabeGadaaakeaacqWG5bqEdaqhaaWcbaGaemyCaeNaeiilaWIaemOAaOMaeyOeI0IaeGymaedabaGaeGimaadaaaaa@34C7@ are the gap states for the *p*-th and *q*-th rows in *A*(1, *i *- 1) and *B*(1, *j *- 1) on Pi−1,j−10
 MathType@MTEF@5@5@+=feaafiart1ev1aaatCvAUfKttLearuWrP9MDH5MBPbIqV92AaeXatLxBI9gBaebbnrfifHhDYfgasaacH8akY=wiFfYdH8Gipec8Eeeu0xXdbba9frFj0=OqFfea0dXdd9vqai=hGuQ8kuc9pgc9s8qqaq=dirpe0xb9q8qiLsFr0=vr0=vr0dc8meaabaqaciaacaGaaeqabaqabeGadaaakeaacqWGqbaudaqhaaWcbaGaemyAaKMaeyOeI0IaeGymaeJaeiilaWIaemOAaOMaeyOeI0IaeGymaedabaGaeGimaadaaaaa@3642@, respectively. As mentioned above, the gap state is the length of the gap up to the current position. *γ*(*a*, *b*, *x*, *y*) represents whether a gap opens with respect to a pair of rows. Specifically, if *a *is a residue, *x *≥ *y*, and *b *is a null; or if *a *is a null, *x *≤ *y*, and *b *is a residue; then *γ*(*a*, *b*, *x*, *y*) = 1. Otherwise, *γ*(*a*, *b*, *x*, *y*) = 0. In order to evaluate exact gap openings for calculation of each Hi,jk
 MathType@MTEF@5@5@+=feaafiart1ev1aaatCvAUfKttLearuWrP9MDH5MBPbIqV92AaeXatLxBI9gBaebbnrfifHhDYfgasaacH8akY=wiFfYdH8Gipec8Eeeu0xXdbba9frFj0=OqFfea0dXdd9vqai=hGuQ8kuc9pgc9s8qqaq=dirpe0xb9q8qiLsFr0=vr0=vr0dc8meaabaqaciaacaGaaeqabaqabeGadaaakeaacqWGibasdaqhaaWcbaGaemyAaKMaeiilaWIaemOAaOgabaGaem4AaSgaaaaa@32E9@, the gap states must be updated. If *a*_*p*,*i *_is a null, xp,i0=xp,i−1d+1
 MathType@MTEF@5@5@+=feaafiart1ev1aaatCvAUfKttLearuWrP9MDH5MBPbIqV92AaeXatLxBI9gBaebbnrfifHhDYfgasaacH8akY=wiFfYdH8Gipec8Eeeu0xXdbba9frFj0=OqFfea0dXdd9vqai=hGuQ8kuc9pgc9s8qqaq=dirpe0xb9q8qiLsFr0=vr0=vr0dc8meaabaqaciaacaGaaeqabaqabeGadaaakeaacqWG4baEdaqhaaWcbaGaemiCaaNaeiilaWIaemyAaKgabaGaeGimaadaaOGaeyypa0JaemiEaG3aa0baaSqaaiabdchaWjabcYcaSiabdMgaPjabgkHiTiabigdaXaqaaiabdsgaKbaakiabgUcaRiabigdaXaaa@3E48@ where *d *= arg max_1≤*k*≤3 _{Hi,jk
 MathType@MTEF@5@5@+=feaafiart1ev1aaatCvAUfKttLearuWrP9MDH5MBPbIqV92AaeXatLxBI9gBaebbnrfifHhDYfgasaacH8akY=wiFfYdH8Gipec8Eeeu0xXdbba9frFj0=OqFfea0dXdd9vqai=hGuQ8kuc9pgc9s8qqaq=dirpe0xb9q8qiLsFr0=vr0=vr0dc8meaabaqaciaacaGaaeqabaqabeGadaaakeaacqWGibasdaqhaaWcbaGaemyAaKMaeiilaWIaemOAaOgabaGaem4AaSgaaaaa@32E9@}. Otherwise, xp,i0
 MathType@MTEF@5@5@+=feaafiart1ev1aaatCvAUfKttLearuWrP9MDH5MBPbIqV92AaeXatLxBI9gBaebbnrfifHhDYfgasaacH8akY=wiFfYdH8Gipec8Eeeu0xXdbba9frFj0=OqFfea0dXdd9vqai=hGuQ8kuc9pgc9s8qqaq=dirpe0xb9q8qiLsFr0=vr0=vr0dc8meaabaqaciaacaGaaeqabaqabeGadaaakeaacqWG4baEdaqhaaWcbaGaemiCaaNaeiilaWIaemyAaKgabaGaeGimaadaaaaa@32E4@ = 0. The other gap states are calculated in a similar way.

#### Use of generalized profile

Although equations 5 and 6 require *O*(*mn*) computational steps, these steps can be reduced by using a generalized profile [19] and the three-way weighting method [20]. The idea of using the generalized profile is that the same residue types or gap states on a column are treated together. The generalized profile consists of four vectors calculated from each column of a group: frequency, residue profile, and two kinds of static gap profile vectors. These vectors can be obtained in advance of the DP process. The frequency and residue profile vectors are used to calculate *S*(**a**_*i*_, **b**_*j*_), while the static gap profile vectors are necessary to calculate *G*(**a**_*i*_, **b**_*j*_; Pi−1,j−10
 MathType@MTEF@5@5@+=feaafiart1ev1aaatCvAUfKttLearuWrP9MDH5MBPbIqV92AaeXatLxBI9gBaebbnrfifHhDYfgasaacH8akY=wiFfYdH8Gipec8Eeeu0xXdbba9frFj0=OqFfea0dXdd9vqai=hGuQ8kuc9pgc9s8qqaq=dirpe0xb9q8qiLsFr0=vr0=vr0dc8meaabaqaciaacaGaaeqabaqabeGadaaakeaacqWGqbaudaqhaaWcbaGaemyAaKMaeyOeI0IaeGymaeJaeiilaWIaemOAaOMaeyOeI0IaeGymaedabaGaeGimaadaaaaa@3642@).

With the frequency and residue profile vectors, *S*(**a**_*i*_, **b**_*j*_) is calculated by

**Figure 1:**
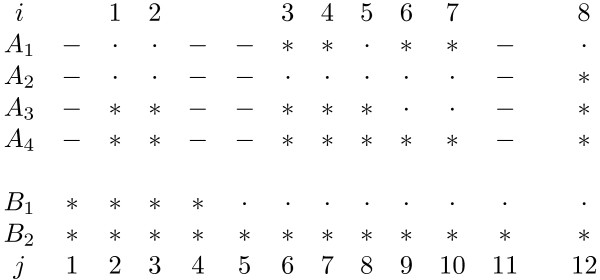
**Example of gap extension penalty calculation**. This figure shows an example of columns **a**_8 _and **b**_12 _being aligned. '*', '·', and '-' denote a residue, a static null, and a dynamic null, respectively. We assume that piecewise linear gap cost *g*(*x*) is max_*k *= 1,2_{-(*u*_*k*_*x *+ *v*_*k*_)} and critical gap length *x*_*c *_(= ⌊(*v*_2 _- *v*_1_)/(*u*_1 _- *u*_2_)⌋) is 4. Gap extension penalty is *u*_1 _if *x *≤ 4, otherwise *u*_2_. Running gap profile vectors S^a8
 MathType@MTEF@5@5@+=feaafiart1ev1aaatCvAUfKttLearuWrP9MDH5MBPbIqV92AaeXatLxBI9gBaebbnrfifHhDYfgasaacH8akY=wiFfYdH8Gipec8Eeeu0xXdbba9frFj0=OqFfea0dXdd9vqai=hGuQ8kuc9pgc9s8qqaq=dirpe0xb9q8qiLsFr0=vr0=vr0dc8meaabaqaciaacaGaaeqabaqabeGadaaakeaacuWGtbWugaqcamaaBaaaleaaieqacqWFHbqydaWgaaadbaGaeGioaGdabeaaaSqabaaaaa@309E@ and E^a8+
 MathType@MTEF@5@5@+=feaafiart1ev1aaatCvAUfKttLearuWrP9MDH5MBPbIqV92AaeXatLxBI9gBaebbnrfifHhDYfgasaacH8akY=wiFfYdH8Gipec8Eeeu0xXdbba9frFj0=OqFfea0dXdd9vqai=hGuQ8kuc9pgc9s8qqaq=dirpe0xb9q8qiLsFr0=vr0=vr0dc8meaabaqaciaacaGaaeqabaqabeGadaaakeaacuWGfbqrgaqcamaaDaaaleaaieqacqWFHbqydaWgaaadbaGaeGioaGdabeaaaSqaaiabgUcaRaaaaaa@3165@ are {(1, wA1
 MathType@MTEF@5@5@+=feaafiart1ev1aaatCvAUfKttLearuWrP9MDH5MBPbIqV92AaeXatLxBI9gBaebbnrfifHhDYfgasaacH8akY=wiFfYdH8Gipec8Eeeu0xXdbba9frFj0=OqFfea0dXdd9vqai=hGuQ8kuc9pgc9s8qqaq=dirpe0xb9q8qiLsFr0=vr0=vr0dc8meaabaqaciaacaGaaeqabaqabeGadaaakeaacqWG3bWDdaWgaaWcbaGaemyqae0aaSbaaWqaaiabigdaXaqabaaaleqaaaaa@3082@)} and {(1, wA2
 MathType@MTEF@5@5@+=feaafiart1ev1aaatCvAUfKttLearuWrP9MDH5MBPbIqV92AaeXatLxBI9gBaebbnrfifHhDYfgasaacH8akY=wiFfYdH8Gipec8Eeeu0xXdbba9frFj0=OqFfea0dXdd9vqai=hGuQ8kuc9pgc9s8qqaq=dirpe0xb9q8qiLsFr0=vr0=vr0dc8meaabaqaciaacaGaaeqabaqabeGadaaakeaacqWG3bWDdaWgaaWcbaGaemyqae0aaSbaaWqaaiabikdaYaqabaaaleqaaaaa@3084@ + wA3
 MathType@MTEF@5@5@+=feaafiart1ev1aaatCvAUfKttLearuWrP9MDH5MBPbIqV92AaeXatLxBI9gBaebbnrfifHhDYfgasaacH8akY=wiFfYdH8Gipec8Eeeu0xXdbba9frFj0=OqFfea0dXdd9vqai=hGuQ8kuc9pgc9s8qqaq=dirpe0xb9q8qiLsFr0=vr0=vr0dc8meaabaqaciaacaGaaeqabaqabeGadaaakeaacqWG3bWDdaWgaaWcbaGaemyqae0aaSbaaWqaaiabiodaZaqabaaaleqaaaaa@3086@ + wA4
 MathType@MTEF@5@5@+=feaafiart1ev1aaatCvAUfKttLearuWrP9MDH5MBPbIqV92AaeXatLxBI9gBaebbnrfifHhDYfgasaacH8akY=wiFfYdH8Gipec8Eeeu0xXdbba9frFj0=OqFfea0dXdd9vqai=hGuQ8kuc9pgc9s8qqaq=dirpe0xb9q8qiLsFr0=vr0=vr0dc8meaabaqaciaacaGaaeqabaqabeGadaaakeaacqWG3bWDdaWgaaWcbaGaemyqae0aaSbaaWqaaiabisda0aqabaaaleqaaaaa@3088@), (3, wA2
 MathType@MTEF@5@5@+=feaafiart1ev1aaatCvAUfKttLearuWrP9MDH5MBPbIqV92AaeXatLxBI9gBaebbnrfifHhDYfgasaacH8akY=wiFfYdH8Gipec8Eeeu0xXdbba9frFj0=OqFfea0dXdd9vqai=hGuQ8kuc9pgc9s8qqaq=dirpe0xb9q8qiLsFr0=vr0=vr0dc8meaabaqaciaacaGaaeqabaqabeGadaaakeaacqWG3bWDdaWgaaWcbaGaemyqae0aaSbaaWqaaiabikdaYaqabaaaleqaaaaa@3084@ + wA3
 MathType@MTEF@5@5@+=feaafiart1ev1aaatCvAUfKttLearuWrP9MDH5MBPbIqV92AaeXatLxBI9gBaebbnrfifHhDYfgasaacH8akY=wiFfYdH8Gipec8Eeeu0xXdbba9frFj0=OqFfea0dXdd9vqai=hGuQ8kuc9pgc9s8qqaq=dirpe0xb9q8qiLsFr0=vr0=vr0dc8meaabaqaciaacaGaaeqabaqabeGadaaakeaacqWG3bWDdaWgaaWcbaGaemyqae0aaSbaaWqaaiabiodaZaqabaaaleqaaaaa@3086@), (11, wA2
 MathType@MTEF@5@5@+=feaafiart1ev1aaatCvAUfKttLearuWrP9MDH5MBPbIqV92AaeXatLxBI9gBaebbnrfifHhDYfgasaacH8akY=wiFfYdH8Gipec8Eeeu0xXdbba9frFj0=OqFfea0dXdd9vqai=hGuQ8kuc9pgc9s8qqaq=dirpe0xb9q8qiLsFr0=vr0=vr0dc8meaabaqaciaacaGaaeqabaqabeGadaaakeaacqWG3bWDdaWgaaWcbaGaemyqae0aaSbaaWqaaiabikdaYaqabaaaleqaaaaa@3084@)}, respectively. Dynamic gap information D7,110
 MathType@MTEF@5@5@+=feaafiart1ev1aaatCvAUfKttLearuWrP9MDH5MBPbIqV92AaeXatLxBI9gBaebbnrfifHhDYfgasaacH8akY=wiFfYdH8Gipec8Eeeu0xXdbba9frFj0=OqFfea0dXdd9vqai=hGuQ8kuc9pgc9s8qqaq=dirpe0xb9q8qiLsFr0=vr0=vr0dc8meaabaqaciaacaGaaeqabaqabeGadaaakeaacqWGebardaqhaaWcbaGaeG4naCJaeiilaWIaeGymaeJaeGymaedabaGaeGimaadaaaaa@3294@(*A*) is {(0, 1), (2, 2), (7, 1)}. Segment profile Fa8
 MathType@MTEF@5@5@+=feaafiart1ev1aaatCvAUfKttLearuWrP9MDH5MBPbIqV92AaeXatLxBI9gBaebbnrfifHhDYfgasaacH8akY=wiFfYdH8Gipec8Eeeu0xXdbba9frFj0=OqFfea0dXdd9vqai=hGuQ8kuc9pgc9s8qqaq=dirpe0xb9q8qiLsFr0=vr0=vr0dc8meaabaqaciaacaGaaeqabaqabeGadaaakeaacqWGgbGrdaWgaaWcbaacbeGae8xyae2aaSbaaWqaaiabiIda4aqabaaaleqaaaaa@3074@ is {(1, wA2
 MathType@MTEF@5@5@+=feaafiart1ev1aaatCvAUfKttLearuWrP9MDH5MBPbIqV92AaeXatLxBI9gBaebbnrfifHhDYfgasaacH8akY=wiFfYdH8Gipec8Eeeu0xXdbba9frFj0=OqFfea0dXdd9vqai=hGuQ8kuc9pgc9s8qqaq=dirpe0xb9q8qiLsFr0=vr0=vr0dc8meaabaqaciaacaGaaeqabaqabeGadaaakeaacqWG3bWDdaWgaaWcbaGaemyqae0aaSbaaWqaaiabikdaYaqabaaaleqaaaaa@3084@), (3, wA2
 MathType@MTEF@5@5@+=feaafiart1ev1aaatCvAUfKttLearuWrP9MDH5MBPbIqV92AaeXatLxBI9gBaebbnrfifHhDYfgasaacH8akY=wiFfYdH8Gipec8Eeeu0xXdbba9frFj0=OqFfea0dXdd9vqai=hGuQ8kuc9pgc9s8qqaq=dirpe0xb9q8qiLsFr0=vr0=vr0dc8meaabaqaciaacaGaaeqabaqabeGadaaakeaacqWG3bWDdaWgaaWcbaGaemyqae0aaSbaaWqaaiabikdaYaqabaaaleqaaaaa@3084@ + wA3
 MathType@MTEF@5@5@+=feaafiart1ev1aaatCvAUfKttLearuWrP9MDH5MBPbIqV92AaeXatLxBI9gBaebbnrfifHhDYfgasaacH8akY=wiFfYdH8Gipec8Eeeu0xXdbba9frFj0=OqFfea0dXdd9vqai=hGuQ8kuc9pgc9s8qqaq=dirpe0xb9q8qiLsFr0=vr0=vr0dc8meaabaqaciaacaGaaeqabaqabeGadaaakeaacqWG3bWDdaWgaaWcbaGaemyqae0aaSbaaWqaaiabiodaZaqabaaaleqaaaaa@3086@), (5, wA2
 MathType@MTEF@5@5@+=feaafiart1ev1aaatCvAUfKttLearuWrP9MDH5MBPbIqV92AaeXatLxBI9gBaebbnrfifHhDYfgasaacH8akY=wiFfYdH8Gipec8Eeeu0xXdbba9frFj0=OqFfea0dXdd9vqai=hGuQ8kuc9pgc9s8qqaq=dirpe0xb9q8qiLsFr0=vr0=vr0dc8meaabaqaciaacaGaaeqabaqabeGadaaakeaacqWG3bWDdaWgaaWcbaGaemyqae0aaSbaaWqaaiabikdaYaqabaaaleqaaaaa@3084@ + wA3
 MathType@MTEF@5@5@+=feaafiart1ev1aaatCvAUfKttLearuWrP9MDH5MBPbIqV92AaeXatLxBI9gBaebbnrfifHhDYfgasaacH8akY=wiFfYdH8Gipec8Eeeu0xXdbba9frFj0=OqFfea0dXdd9vqai=hGuQ8kuc9pgc9s8qqaq=dirpe0xb9q8qiLsFr0=vr0=vr0dc8meaabaqaciaacaGaaeqabaqabeGadaaakeaacqWG3bWDdaWgaaWcbaGaemyqae0aaSbaaWqaaiabiodaZaqabaaaleqaaaaa@3086@ + wA4
 MathType@MTEF@5@5@+=feaafiart1ev1aaatCvAUfKttLearuWrP9MDH5MBPbIqV92AaeXatLxBI9gBaebbnrfifHhDYfgasaacH8akY=wiFfYdH8Gipec8Eeeu0xXdbba9frFj0=OqFfea0dXdd9vqai=hGuQ8kuc9pgc9s8qqaq=dirpe0xb9q8qiLsFr0=vr0=vr0dc8meaabaqaciaacaGaaeqabaqabeGadaaakeaacqWG3bWDdaWgaaWcbaGaemyqae0aaSbaaWqaaiabisda0aqabaaaleqaaaaa@3088@)}. Similarly, the profile vectors of *B *are defined: S^b12
 MathType@MTEF@5@5@+=feaafiart1ev1aaatCvAUfKttLearuWrP9MDH5MBPbIqV92AaeXatLxBI9gBaebbnrfifHhDYfgasaacH8akY=wiFfYdH8Gipec8Eeeu0xXdbba9frFj0=OqFfea0dXdd9vqai=hGuQ8kuc9pgc9s8qqaq=dirpe0xb9q8qiLsFr0=vr0=vr0dc8meaabaqaciaacaGaaeqabaqabeGadaaakeaacuWGtbWugaqcamaaBaaaleaaieqacqWFIbGydaWgaaadbaGaeGymaeJaeGOmaidabeaaaSqabaaaaa@3184@ = {(7, wB1
 MathType@MTEF@5@5@+=feaafiart1ev1aaatCvAUfKttLearuWrP9MDH5MBPbIqV92AaeXatLxBI9gBaebbnrfifHhDYfgasaacH8akY=wiFfYdH8Gipec8Eeeu0xXdbba9frFj0=OqFfea0dXdd9vqai=hGuQ8kuc9pgc9s8qqaq=dirpe0xb9q8qiLsFr0=vr0=vr0dc8meaabaqaciaacaGaaeqabaqabeGadaaakeaacqWG3bWDdaWgaaWcbaGaemOqai0aaSbaaWqaaiabigdaXaqabaaaleqaaaaa@3084@)}, E^b12+
 MathType@MTEF@5@5@+=feaafiart1ev1aaatCvAUfKttLearuWrP9MDH5MBPbIqV92AaeXatLxBI9gBaebbnrfifHhDYfgasaacH8akY=wiFfYdH8Gipec8Eeeu0xXdbba9frFj0=OqFfea0dXdd9vqai=hGuQ8kuc9pgc9s8qqaq=dirpe0xb9q8qiLsFr0=vr0=vr0dc8meaabaqaciaacaGaaeqabaqabeGadaaakeaacuWGfbqrgaqcamaaDaaaleaaieqacqWFIbGydaWgaaadbaGaeGymaeJaeGOmaidabeaaaSqaaiabgUcaRaaaaaa@324B@ = {(0, wB2
 MathType@MTEF@5@5@+=feaafiart1ev1aaatCvAUfKttLearuWrP9MDH5MBPbIqV92AaeXatLxBI9gBaebbnrfifHhDYfgasaacH8akY=wiFfYdH8Gipec8Eeeu0xXdbba9frFj0=OqFfea0dXdd9vqai=hGuQ8kuc9pgc9s8qqaq=dirpe0xb9q8qiLsFr0=vr0=vr0dc8meaabaqaciaacaGaaeqabaqabeGadaaakeaacqWG3bWDdaWgaaWcbaGaemOqai0aaSbaaWqaaiabikdaYaqabaaaleqaaaaa@3086@)}, D7,110
 MathType@MTEF@5@5@+=feaafiart1ev1aaatCvAUfKttLearuWrP9MDH5MBPbIqV92AaeXatLxBI9gBaebbnrfifHhDYfgasaacH8akY=wiFfYdH8Gipec8Eeeu0xXdbba9frFj0=OqFfea0dXdd9vqai=hGuQ8kuc9pgc9s8qqaq=dirpe0xb9q8qiLsFr0=vr0=vr0dc8meaabaqaciaacaGaaeqabaqabeGadaaakeaacqWGebardaqhaaWcbaGaeG4naCJaeiilaWIaeGymaeJaeGymaedabaGaeGimaadaaaaa@3294@(*B*) is empty, and Fb12
 MathType@MTEF@5@5@+=feaafiart1ev1aaatCvAUfKttLearuWrP9MDH5MBPbIqV92AaeXatLxBI9gBaebbnrfifHhDYfgasaacH8akY=wiFfYdH8Gipec8Eeeu0xXdbba9frFj0=OqFfea0dXdd9vqai=hGuQ8kuc9pgc9s8qqaq=dirpe0xb9q8qiLsFr0=vr0=vr0dc8meaabaqaciaacaGaaeqabaqabeGadaaakeaacqWGgbGrdaWgaaWcbaacbeGae8Nyai2aaSbaaWqaaiabigdaXiabikdaYaqabaaaleqaaaaa@315A@ = {(1, 0), (9, wB2
 MathType@MTEF@5@5@+=feaafiart1ev1aaatCvAUfKttLearuWrP9MDH5MBPbIqV92AaeXatLxBI9gBaebbnrfifHhDYfgasaacH8akY=wiFfYdH8Gipec8Eeeu0xXdbba9frFj0=OqFfea0dXdd9vqai=hGuQ8kuc9pgc9s8qqaq=dirpe0xb9q8qiLsFr0=vr0=vr0dc8meaabaqaciaacaGaaeqabaqabeGadaaakeaacqWG3bWDdaWgaaWcbaGaemOqai0aaSbaaWqaaiabikdaYaqabaaaleqaaaaa@3086@)}. In what follows, we consider the non-trivial calculation of the gap extension penalty with respect to the gap of *B*_1_, the target gap. By using S^b11
 MathType@MTEF@5@5@+=feaafiart1ev1aaatCvAUfKttLearuWrP9MDH5MBPbIqV92AaeXatLxBI9gBaebbnrfifHhDYfgasaacH8akY=wiFfYdH8Gipec8Eeeu0xXdbba9frFj0=OqFfea0dXdd9vqai=hGuQ8kuc9pgc9s8qqaq=dirpe0xb9q8qiLsFr0=vr0=vr0dc8meaabaqaciaacaGaaeqabaqabeGadaaakeaacuWGtbWugaqcamaaBaaaleaaieqacqWFIbGydaWgaaadbaGaeGymaeJaeGymaedabeaaaSqabaaaaa@3182@ and D7,110
 MathType@MTEF@5@5@+=feaafiart1ev1aaatCvAUfKttLearuWrP9MDH5MBPbIqV92AaeXatLxBI9gBaebbnrfifHhDYfgasaacH8akY=wiFfYdH8Gipec8Eeeu0xXdbba9frFj0=OqFfea0dXdd9vqai=hGuQ8kuc9pgc9s8qqaq=dirpe0xb9q8qiLsFr0=vr0=vr0dc8meaabaqaciaacaGaaeqabaqabeGadaaakeaacqWGebardaqhaaWcbaGaeG4naCJaeiilaWIaeGymaeJaeGymaedabaGaeGimaadaaaaa@3294@(*A*), we find that the two dynamic gaps specified by (2, 2) and (7, 1) in D7,110
 MathType@MTEF@5@5@+=feaafiart1ev1aaatCvAUfKttLearuWrP9MDH5MBPbIqV92AaeXatLxBI9gBaebbnrfifHhDYfgasaacH8akY=wiFfYdH8Gipec8Eeeu0xXdbba9frFj0=OqFfea0dXdd9vqai=hGuQ8kuc9pgc9s8qqaq=dirpe0xb9q8qiLsFr0=vr0=vr0dc8meaabaqaciaacaGaaeqabaqabeGadaaakeaacqWGebardaqhaaWcbaGaeG4naCJaeiilaWIaeGymaeJaeGymaedabaGaeGimaadaaaaa@3294@(*A*) are partially and completely aligned with the target gap, respectively. Consequently, the total number of nulls aligned with null columns of dynamic gaps to be removed is 2. Therefore, the number of columns of *B*_1 _is 5(= 7 - 2). By subtracting 5 from 8 (the end position of the segment), the starting position of *A*, 3, is obtained. Then, the gap extension penalty with respect to the gap of *B*_1 _is wB1
 MathType@MTEF@5@5@+=feaafiart1ev1aaatCvAUfKttLearuWrP9MDH5MBPbIqV92AaeXatLxBI9gBaebbnrfifHhDYfgasaacH8akY=wiFfYdH8Gipec8Eeeu0xXdbba9frFj0=OqFfea0dXdd9vqai=hGuQ8kuc9pgc9s8qqaq=dirpe0xb9q8qiLsFr0=vr0=vr0dc8meaabaqaciaacaGaaeqabaqabeGadaaakeaacqWG3bWDdaWgaaWcbaGaemOqai0aaSbaaWqaaiabigdaXaqabaaaleqaaaaa@3084@ (*F*_1_·*u*_1 _+ *F*_2_·*u*_2_) where *F*_1 _= wA2
 MathType@MTEF@5@5@+=feaafiart1ev1aaatCvAUfKttLearuWrP9MDH5MBPbIqV92AaeXatLxBI9gBaebbnrfifHhDYfgasaacH8akY=wiFfYdH8Gipec8Eeeu0xXdbba9frFj0=OqFfea0dXdd9vqai=hGuQ8kuc9pgc9s8qqaq=dirpe0xb9q8qiLsFr0=vr0=vr0dc8meaabaqaciaacaGaaeqabaqabeGadaaakeaacqWG3bWDdaWgaaWcbaGaemyqae0aaSbaaWqaaiabikdaYaqabaaaleqaaaaa@3084@ + wA3
 MathType@MTEF@5@5@+=feaafiart1ev1aaatCvAUfKttLearuWrP9MDH5MBPbIqV92AaeXatLxBI9gBaebbnrfifHhDYfgasaacH8akY=wiFfYdH8Gipec8Eeeu0xXdbba9frFj0=OqFfea0dXdd9vqai=hGuQ8kuc9pgc9s8qqaq=dirpe0xb9q8qiLsFr0=vr0=vr0dc8meaabaqaciaacaGaaeqabaqabeGadaaakeaacqWG3bWDdaWgaaWcbaGaemyqae0aaSbaaWqaaiabiodaZaqabaaaleqaaaaa@3086@ and *F*_2 _= wA4
 MathType@MTEF@5@5@+=feaafiart1ev1aaatCvAUfKttLearuWrP9MDH5MBPbIqV92AaeXatLxBI9gBaebbnrfifHhDYfgasaacH8akY=wiFfYdH8Gipec8Eeeu0xXdbba9frFj0=OqFfea0dXdd9vqai=hGuQ8kuc9pgc9s8qqaq=dirpe0xb9q8qiLsFr0=vr0=vr0dc8meaabaqaciaacaGaaeqabaqabeGadaaakeaacqWG3bWDdaWgaaWcbaGaemyqae0aaSbaaWqaaiabisda0aqabaaaleqaaaaa@3088@. Note that *A*_1 _is not involved in the gap extension penalty because *a*_1,8 _is a null.

S(ai,bj)=∑r∈Σ*fai,r⋅pbj,r=∑r∈Σ*pai,r⋅fbj,r     (7)
 MathType@MTEF@5@5@+=feaafiart1ev1aaatCvAUfKttLearuWrP9MDH5MBPbIqV92AaeXatLxBI9gBaebbnrfifHhDYfgasaacH8akY=wiFfYdH8Gipec8Eeeu0xXdbba9frFj0=OqFfea0dXdd9vqai=hGuQ8kuc9pgc9s8qqaq=dirpe0xb9q8qiLsFr0=vr0=vr0dc8meaabaqaciaacaGaaeqabaqabeGadaaakeaacqWGtbWucqGGOaakieqacqWFHbqydaWgaaWcbaGaemyAaKgabeaakiabcYcaSiab=jgaInaaBaaaleaacqWGQbGAaeqaaOGaeiykaKIaeyypa0ZaaabuaeaacqWGMbGzdaWgaaWcbaGae8xyae2aaSbaaWqaaiabdMgaPbqabaWccqGGSaalcqWGYbGCaeqaaaqaaiabdkhaYjabgIGiolabfo6atjabcQcaQaqab0GaeyyeIuoakiabgwSixlabdchaWnaaBaaaleaacqWFIbGydaWgaaadbaGaemOAaOgabeaaliabcYcaSiabdkhaYbqabaGccqGH9aqpdaaeqbqaaiabdchaWnaaBaaaleaacqWFHbqydaWgaaadbaGaemyAaKgabeaaliabcYcaSiabdkhaYbqabaaabaGaemOCaiNaeyicI4Saeu4OdmLaeiOkaOcabeqdcqGHris5aOGaeyyXICTaemOzay2aaSbaaSqaaiab=jgaInaaBaaameaacqWGQbGAaeqaaSGaeiilaWIaemOCaihabeaakiaaxMaacaWLjaWaaeWaaeaacqaI3aWnaiaawIcacaGLPaaaaaa@6A5E@

where residue frequency fai,r
 MathType@MTEF@5@5@+=feaafiart1ev1aaatCvAUfKttLearuWrP9MDH5MBPbIqV92AaeXatLxBI9gBaebbnrfifHhDYfgasaacH8akY=wiFfYdH8Gipec8Eeeu0xXdbba9frFj0=OqFfea0dXdd9vqai=hGuQ8kuc9pgc9s8qqaq=dirpe0xb9q8qiLsFr0=vr0=vr0dc8meaabaqaciaacaGaaeqabaqabeGadaaakeaacqWGMbGzdaWgaaWcbaacbeGae8xyae2aaSbaaWqaaiabdMgaPbqabaWccqGGSaalcqWGYbGCaeqaaaaa@335E@ is the weighted frequency of occurrence of residue type *r *(including null) on column **a**_*i*_, and residue profile pbj,r=∑t∈Σ*fbj,r⋅s(r,t)
 MathType@MTEF@5@5@+=feaafiart1ev1aaatCvAUfKttLearuWrP9MDH5MBPbIqV92AaeXatLxBI9gBaebbnrfifHhDYfgasaacH8akY=wiFfYdH8Gipec8Eeeu0xXdbba9frFj0=OqFfea0dXdd9vqai=hGuQ8kuc9pgc9s8qqaq=dirpe0xb9q8qiLsFr0=vr0=vr0dc8meaabaqaciaacaGaaeqabaqabeGadaaakeaacqWGWbaCdaWgaaWcbaacbeGae8Nyai2aaSbaaWqaaiabdQgaQbqabaWccqGGSaalcqWGYbGCaeqaaOGaeyypa0ZaaabeaeaacqWGMbGzdaWgaaWcbaGae8Nyai2aaSbaaWqaaiabdQgaQbqabaWccqGGSaalcqWGYbGCaeqaaaqaaiabdsha0jabgIGiolabfo6atjabcQcaQaqab0GaeyyeIuoakiabgwSixlabdohaZjabcIcaOiabdkhaYjabcYcaSiabdsha0jabcMcaPaaa@4B92@. Both pai,r
 MathType@MTEF@5@5@+=feaafiart1ev1aaatCvAUfKttLearuWrP9MDH5MBPbIqV92AaeXatLxBI9gBaebbnrfifHhDYfgasaacH8akY=wiFfYdH8Gipec8Eeeu0xXdbba9frFj0=OqFfea0dXdd9vqai=hGuQ8kuc9pgc9s8qqaq=dirpe0xb9q8qiLsFr0=vr0=vr0dc8meaabaqaciaacaGaaeqabaqabeGadaaakeaacqWGWbaCdaWgaaWcbaacbeGae8xyae2aaSbaaWqaaiabdMgaPbqabaWccqGGSaalcqWGYbGCaeqaaaaa@3372@ and fbj,r
 MathType@MTEF@5@5@+=feaafiart1ev1aaatCvAUfKttLearuWrP9MDH5MBPbIqV92AaeXatLxBI9gBaebbnrfifHhDYfgasaacH8akY=wiFfYdH8Gipec8Eeeu0xXdbba9frFj0=OqFfea0dXdd9vqai=hGuQ8kuc9pgc9s8qqaq=dirpe0xb9q8qiLsFr0=vr0=vr0dc8meaabaqaciaacaGaaeqabaqabeGadaaakeaacqWGMbGzdaWgaaWcbaacbeGae8Nyai2aaSbaaWqaaiabdQgaQbqabaWccqGGSaalcqWGYbGCaeqaaaaa@3362@ are defined in the same way. The right hand side of equation 7 requires *O*(|Σ*|), because each frequency and residue profile vector consists of |Σ*| values. When *m *× *n *is sufficiently large, the computation time can be considerably reduced. Although *S*(**a**_*i*_, **b**_*j*_) is easy to calculate, the profile-based calculation of *G*(**a**_*i*_, **b**_*j*_; Pi−1,j−10
 MathType@MTEF@5@5@+=feaafiart1ev1aaatCvAUfKttLearuWrP9MDH5MBPbIqV92AaeXatLxBI9gBaebbnrfifHhDYfgasaacH8akY=wiFfYdH8Gipec8Eeeu0xXdbba9frFj0=OqFfea0dXdd9vqai=hGuQ8kuc9pgc9s8qqaq=dirpe0xb9q8qiLsFr0=vr0=vr0dc8meaabaqaciaacaGaaeqabaqabeGadaaakeaacqWGqbaudaqhaaWcbaGaemyAaKMaeyOeI0IaeGymaeJaeiilaWIaemOAaOMaeyOeI0IaeGymaedabaGaeGimaadaaaaa@3642@) is somewhat complicated, because, in addition to static gaps, dynamic gaps must be considered explicitly. A static gap consists of consecutive static nulls that already exist in each group, while a dynamic gap denotes a run of dynamic nulls that are inserted into each group during the DP process. Note that we distinguish static and dynamic gaps for convenience of the description of our algorithm, while they contribute to the total alignment score in the same way. Let us consider an example of the gap opening penalty calculation when **a**_8 _is aligned with **b**_12 _(Figure 1). To keep the discussion simple, we consider *A*_1 _and *B*_2 _only. From simple observation, we find that a gap between *A*_1 _and *B*_2 _has already opened before **a**_8 _is aligned with **b**_12_. However, if the gap opening penalty were calculated using the static gap states only, a gap opening would be detected wrongly, because *a*_1,8 _is a null, *b*_2,12 _is a residue, and *x*_1,7 _= 0 ≤ *y*_2,11 _= 0 where *x*_1,7 _and *y*_2,11 _are the static gap states of *a*_1,7 _and *b*_2,11_, respectively. For correct detection of gap opening, we need 'running gap states', each of which represents the sum of the numbers of consecutive static and dynamic nulls up to the current position. For the example shown in Figure 1, the running gap state for *A*_2 _at column position **a**_7 _is 11, which is composed of 7 static nulls and 4 dynamic nulls.

Static gap states are compactly represented as a static gap profile. A static gap profile is obtained by gathering static gap states with the same values at a column. More specifically, the static gap profile at a column **c**_*k *_consists of two vectors Eck
 MathType@MTEF@5@5@+=feaafiart1ev1aaatCvAUfKttLearuWrP9MDH5MBPbIqV92AaeXatLxBI9gBaebbnrfifHhDYfgasaacH8akY=wiFfYdH8Gipec8Eeeu0xXdbba9frFj0=OqFfea0dXdd9vqai=hGuQ8kuc9pgc9s8qqaq=dirpe0xb9q8qiLsFr0=vr0=vr0dc8meaabaqaciaacaGaaeqabaqabeGadaaakeaacqWGfbqrdaWgaaWcbaacbeGae83yam2aaSbaaWqaaiabdUgaRbqabaaaleqaaaaa@30D7@ and Sck
 MathType@MTEF@5@5@+=feaafiart1ev1aaatCvAUfKttLearuWrP9MDH5MBPbIqV92AaeXatLxBI9gBaebbnrfifHhDYfgasaacH8akY=wiFfYdH8Gipec8Eeeu0xXdbba9frFj0=OqFfea0dXdd9vqai=hGuQ8kuc9pgc9s8qqaq=dirpe0xb9q8qiLsFr0=vr0=vr0dc8meaabaqaciaacaGaaeqabaqabeGadaaakeaacqWGtbWudaWgaaWcbaacbeGae83yam2aaSbaaWqaaiabdUgaRbqabaaaleqaaaaa@30F3@. Each element of both vectors has the same form: {(*g*, *f*)} where *g *is the gap state of previous column **c**_*k*-1 _and *f *is the weighted frequency of the occurrence of rows whose element on **c**_*k *_is either a residue or null depending on Eck
 MathType@MTEF@5@5@+=feaafiart1ev1aaatCvAUfKttLearuWrP9MDH5MBPbIqV92AaeXatLxBI9gBaebbnrfifHhDYfgasaacH8akY=wiFfYdH8Gipec8Eeeu0xXdbba9frFj0=OqFfea0dXdd9vqai=hGuQ8kuc9pgc9s8qqaq=dirpe0xb9q8qiLsFr0=vr0=vr0dc8meaabaqaciaacaGaaeqabaqabeGadaaakeaacqWGfbqrdaWgaaWcbaacbeGae83yam2aaSbaaWqaaiabdUgaRbqabaaaleqaaaaa@30D7@ or Sck
 MathType@MTEF@5@5@+=feaafiart1ev1aaatCvAUfKttLearuWrP9MDH5MBPbIqV92AaeXatLxBI9gBaebbnrfifHhDYfgasaacH8akY=wiFfYdH8Gipec8Eeeu0xXdbba9frFj0=OqFfea0dXdd9vqai=hGuQ8kuc9pgc9s8qqaq=dirpe0xb9q8qiLsFr0=vr0=vr0dc8meaabaqaciaacaGaaeqabaqabeGadaaakeaacqWGtbWudaWgaaWcbaacbeGae83yam2aaSbaaWqaaiabdUgaRbqabaaaleqaaaaa@30F3@, respectively. Although Eck
 MathType@MTEF@5@5@+=feaafiart1ev1aaatCvAUfKttLearuWrP9MDH5MBPbIqV92AaeXatLxBI9gBaebbnrfifHhDYfgasaacH8akY=wiFfYdH8Gipec8Eeeu0xXdbba9frFj0=OqFfea0dXdd9vqai=hGuQ8kuc9pgc9s8qqaq=dirpe0xb9q8qiLsFr0=vr0=vr0dc8meaabaqaciaacaGaaeqabaqabeGadaaakeaacqWGfbqrdaWgaaWcbaacbeGae83yam2aaSbaaWqaaiabdUgaRbqabaaaleqaaaaa@30D7@ itself may be used to calculate gap opening penalties, its accumulated form, Eck+
 MathType@MTEF@5@5@+=feaafiart1ev1aaatCvAUfKttLearuWrP9MDH5MBPbIqV92AaeXatLxBI9gBaebbnrfifHhDYfgasaacH8akY=wiFfYdH8Gipec8Eeeu0xXdbba9frFj0=OqFfea0dXdd9vqai=hGuQ8kuc9pgc9s8qqaq=dirpe0xb9q8qiLsFr0=vr0=vr0dc8meaabaqaciaacaGaaeqabaqabeGadaaakeaacqWGfbqrdaqhaaWcbaacbeGae83yam2aaSbaaWqaaiabdUgaRbqabaaaleaacqGHRaWkaaaaaa@31BA@, is more convenient to reduce the computation time. If an element in Eck+
 MathType@MTEF@5@5@+=feaafiart1ev1aaatCvAUfKttLearuWrP9MDH5MBPbIqV92AaeXatLxBI9gBaebbnrfifHhDYfgasaacH8akY=wiFfYdH8Gipec8Eeeu0xXdbba9frFj0=OqFfea0dXdd9vqai=hGuQ8kuc9pgc9s8qqaq=dirpe0xb9q8qiLsFr0=vr0=vr0dc8meaabaqaciaacaGaaeqabaqabeGadaaakeaacqWGfbqrdaqhaaWcbaacbeGae83yam2aaSbaaWqaaiabdUgaRbqabaaaleaacqGHRaWkaaaaaa@31BA@ is (*g*, *f*^+^), *f*^+ ^represents the weighted frequency of the occurrence of rows whose gap states are not less than *g*. For the example shown in Figure 1, Ea8
 MathType@MTEF@5@5@+=feaafiart1ev1aaatCvAUfKttLearuWrP9MDH5MBPbIqV92AaeXatLxBI9gBaebbnrfifHhDYfgasaacH8akY=wiFfYdH8Gipec8Eeeu0xXdbba9frFj0=OqFfea0dXdd9vqai=hGuQ8kuc9pgc9s8qqaq=dirpe0xb9q8qiLsFr0=vr0=vr0dc8meaabaqaciaacaGaaeqabaqabeGadaaakeaacqWGfbqrdaWgaaWcbaacbeGae8xyae2aaSbaaWqaaiabiIda4aqabaaaleqaaaaa@3072@, Ea8+
 MathType@MTEF@5@5@+=feaafiart1ev1aaatCvAUfKttLearuWrP9MDH5MBPbIqV92AaeXatLxBI9gBaebbnrfifHhDYfgasaacH8akY=wiFfYdH8Gipec8Eeeu0xXdbba9frFj0=OqFfea0dXdd9vqai=hGuQ8kuc9pgc9s8qqaq=dirpe0xb9q8qiLsFr0=vr0=vr0dc8meaabaqaciaacaGaaeqabaqabeGadaaakeaacqWGfbqrdaqhaaWcbaacbeGae8xyae2aaSbaaWqaaiabiIda4aqabaaaleaacqGHRaWkaaaaaa@3155@, and Sa8
 MathType@MTEF@5@5@+=feaafiart1ev1aaatCvAUfKttLearuWrP9MDH5MBPbIqV92AaeXatLxBI9gBaebbnrfifHhDYfgasaacH8akY=wiFfYdH8Gipec8Eeeu0xXdbba9frFj0=OqFfea0dXdd9vqai=hGuQ8kuc9pgc9s8qqaq=dirpe0xb9q8qiLsFr0=vr0=vr0dc8meaabaqaciaacaGaaeqabaqabeGadaaakeaacqWGtbWudaWgaaWcbaacbeGae8xyae2aaSbaaWqaaiabiIda4aqabaaaleqaaaaa@308E@ are {(0, wA4
 MathType@MTEF@5@5@+=feaafiart1ev1aaatCvAUfKttLearuWrP9MDH5MBPbIqV92AaeXatLxBI9gBaebbnrfifHhDYfgasaacH8akY=wiFfYdH8Gipec8Eeeu0xXdbba9frFj0=OqFfea0dXdd9vqai=hGuQ8kuc9pgc9s8qqaq=dirpe0xb9q8qiLsFr0=vr0=vr0dc8meaabaqaciaacaGaaeqabaqabeGadaaakeaacqWG3bWDdaWgaaWcbaGaemyqae0aaSbaaWqaaiabisda0aqabaaaleqaaaaa@3088@), (2, wA3
 MathType@MTEF@5@5@+=feaafiart1ev1aaatCvAUfKttLearuWrP9MDH5MBPbIqV92AaeXatLxBI9gBaebbnrfifHhDYfgasaacH8akY=wiFfYdH8Gipec8Eeeu0xXdbba9frFj0=OqFfea0dXdd9vqai=hGuQ8kuc9pgc9s8qqaq=dirpe0xb9q8qiLsFr0=vr0=vr0dc8meaabaqaciaacaGaaeqabaqabeGadaaakeaacqWG3bWDdaWgaaWcbaGaemyqae0aaSbaaWqaaiabiodaZaqabaaaleqaaaaa@3086@), (7, wA2
 MathType@MTEF@5@5@+=feaafiart1ev1aaatCvAUfKttLearuWrP9MDH5MBPbIqV92AaeXatLxBI9gBaebbnrfifHhDYfgasaacH8akY=wiFfYdH8Gipec8Eeeu0xXdbba9frFj0=OqFfea0dXdd9vqai=hGuQ8kuc9pgc9s8qqaq=dirpe0xb9q8qiLsFr0=vr0=vr0dc8meaabaqaciaacaGaaeqabaqabeGadaaakeaacqWG3bWDdaWgaaWcbaGaemyqae0aaSbaaWqaaiabikdaYaqabaaaleqaaaaa@3084@)}, {(0, wA4
 MathType@MTEF@5@5@+=feaafiart1ev1aaatCvAUfKttLearuWrP9MDH5MBPbIqV92AaeXatLxBI9gBaebbnrfifHhDYfgasaacH8akY=wiFfYdH8Gipec8Eeeu0xXdbba9frFj0=OqFfea0dXdd9vqai=hGuQ8kuc9pgc9s8qqaq=dirpe0xb9q8qiLsFr0=vr0=vr0dc8meaabaqaciaacaGaaeqabaqabeGadaaakeaacqWG3bWDdaWgaaWcbaGaemyqae0aaSbaaWqaaiabisda0aqabaaaleqaaaaa@3088@ + wA3
 MathType@MTEF@5@5@+=feaafiart1ev1aaatCvAUfKttLearuWrP9MDH5MBPbIqV92AaeXatLxBI9gBaebbnrfifHhDYfgasaacH8akY=wiFfYdH8Gipec8Eeeu0xXdbba9frFj0=OqFfea0dXdd9vqai=hGuQ8kuc9pgc9s8qqaq=dirpe0xb9q8qiLsFr0=vr0=vr0dc8meaabaqaciaacaGaaeqabaqabeGadaaakeaacqWG3bWDdaWgaaWcbaGaemyqae0aaSbaaWqaaiabiodaZaqabaaaleqaaaaa@3086@ + wA2
 MathType@MTEF@5@5@+=feaafiart1ev1aaatCvAUfKttLearuWrP9MDH5MBPbIqV92AaeXatLxBI9gBaebbnrfifHhDYfgasaacH8akY=wiFfYdH8Gipec8Eeeu0xXdbba9frFj0=OqFfea0dXdd9vqai=hGuQ8kuc9pgc9s8qqaq=dirpe0xb9q8qiLsFr0=vr0=vr0dc8meaabaqaciaacaGaaeqabaqabeGadaaakeaacqWG3bWDdaWgaaWcbaGaemyqae0aaSbaaWqaaiabikdaYaqabaaaleqaaaaa@3084@), (2, wA3
 MathType@MTEF@5@5@+=feaafiart1ev1aaatCvAUfKttLearuWrP9MDH5MBPbIqV92AaeXatLxBI9gBaebbnrfifHhDYfgasaacH8akY=wiFfYdH8Gipec8Eeeu0xXdbba9frFj0=OqFfea0dXdd9vqai=hGuQ8kuc9pgc9s8qqaq=dirpe0xb9q8qiLsFr0=vr0=vr0dc8meaabaqaciaacaGaaeqabaqabeGadaaakeaacqWG3bWDdaWgaaWcbaGaemyqae0aaSbaaWqaaiabiodaZaqabaaaleqaaaaa@3086@ + wA2
 MathType@MTEF@5@5@+=feaafiart1ev1aaatCvAUfKttLearuWrP9MDH5MBPbIqV92AaeXatLxBI9gBaebbnrfifHhDYfgasaacH8akY=wiFfYdH8Gipec8Eeeu0xXdbba9frFj0=OqFfea0dXdd9vqai=hGuQ8kuc9pgc9s8qqaq=dirpe0xb9q8qiLsFr0=vr0=vr0dc8meaabaqaciaacaGaaeqabaqabeGadaaakeaacqWG3bWDdaWgaaWcbaGaemyqae0aaSbaaWqaaiabikdaYaqabaaaleqaaaaa@3084@), (7, wA2
 MathType@MTEF@5@5@+=feaafiart1ev1aaatCvAUfKttLearuWrP9MDH5MBPbIqV92AaeXatLxBI9gBaebbnrfifHhDYfgasaacH8akY=wiFfYdH8Gipec8Eeeu0xXdbba9frFj0=OqFfea0dXdd9vqai=hGuQ8kuc9pgc9s8qqaq=dirpe0xb9q8qiLsFr0=vr0=vr0dc8meaabaqaciaacaGaaeqabaqabeGadaaakeaacqWG3bWDdaWgaaWcbaGaemyqae0aaSbaaWqaaiabikdaYaqabaaaleqaaaaa@3084@)}, and {(0, wA1
 MathType@MTEF@5@5@+=feaafiart1ev1aaatCvAUfKttLearuWrP9MDH5MBPbIqV92AaeXatLxBI9gBaebbnrfifHhDYfgasaacH8akY=wiFfYdH8Gipec8Eeeu0xXdbba9frFj0=OqFfea0dXdd9vqai=hGuQ8kuc9pgc9s8qqaq=dirpe0xb9q8qiLsFr0=vr0=vr0dc8meaabaqaciaacaGaaeqabaqabeGadaaakeaacqWG3bWDdaWgaaWcbaGaemyqae0aaSbaaWqaaiabigdaXaqabaaaleqaaaaa@3082@)}, respectively. Since all gap states are different in the worst case, the total number of elements of Eck+
 MathType@MTEF@5@5@+=feaafiart1ev1aaatCvAUfKttLearuWrP9MDH5MBPbIqV92AaeXatLxBI9gBaebbnrfifHhDYfgasaacH8akY=wiFfYdH8Gipec8Eeeu0xXdbba9frFj0=OqFfea0dXdd9vqai=hGuQ8kuc9pgc9s8qqaq=dirpe0xb9q8qiLsFr0=vr0=vr0dc8meaabaqaciaacaGaaeqabaqabeGadaaakeaacqWGfbqrdaqhaaWcbaacbeGae83yam2aaSbaaWqaaiabdUgaRbqabaaaleaacqGHRaWkaaaaaa@31BA@ and Sck
 MathType@MTEF@5@5@+=feaafiart1ev1aaatCvAUfKttLearuWrP9MDH5MBPbIqV92AaeXatLxBI9gBaebbnrfifHhDYfgasaacH8akY=wiFfYdH8Gipec8Eeeu0xXdbba9frFj0=OqFfea0dXdd9vqai=hGuQ8kuc9pgc9s8qqaq=dirpe0xb9q8qiLsFr0=vr0=vr0dc8meaabaqaciaacaGaaeqabaqabeGadaaakeaacqWGtbWudaWgaaWcbaacbeGae83yam2aaSbaaWqaaiabdUgaRbqabaaaleqaaaaa@30F3@ is at most the number of sequences in the group.

Like a static gap profile, a running gap profile can represent running gap states compactly. In order to obtain a running gap profile, another data structure called a gap mediation profile is required. A gap mediation profile is defined for each path in the DP process, and records the total number of dynamic nulls inserted into static gaps that are currently open. Each element of the gap mediation profile is expressed as (*s*, *d*), where *s *is the length of a static gap and *d *is the summed length of dynamic gaps inserted within or after the static gap. Let Mi,j0
 MathType@MTEF@5@5@+=feaafiart1ev1aaatCvAUfKttLearuWrP9MDH5MBPbIqV92AaeXatLxBI9gBaebbnrfifHhDYfgasaacH8akY=wiFfYdH8Gipec8Eeeu0xXdbba9frFj0=OqFfea0dXdd9vqai=hGuQ8kuc9pgc9s8qqaq=dirpe0xb9q8qiLsFr0=vr0=vr0dc8meaabaqaciaacaGaaeqabaqabeGadaaakeaacqWGnbqtdaqhaaWcbaGaemyAaKMaeiilaWIaemOAaOgabaGaeGimaadaaaaa@3282@(*A*) be the gap mediation profile of group *A *at the DP node (*i*, *j*)·Mi,j0
 MathType@MTEF@5@5@+=feaafiart1ev1aaatCvAUfKttLearuWrP9MDH5MBPbIqV92AaeXatLxBI9gBaebbnrfifHhDYfgasaacH8akY=wiFfYdH8Gipec8Eeeu0xXdbba9frFj0=OqFfea0dXdd9vqai=hGuQ8kuc9pgc9s8qqaq=dirpe0xb9q8qiLsFr0=vr0=vr0dc8meaabaqaciaacaGaaeqabaqabeGadaaakeaacqWGnbqtdaqhaaWcbaGaemyAaKMaeiilaWIaemOAaOgabaGaeGimaadaaaaa@3282@(*A*) follows a recurrent relation, the initial condition of which is Mi,j0
 MathType@MTEF@5@5@+=feaafiart1ev1aaatCvAUfKttLearuWrP9MDH5MBPbIqV92AaeXatLxBI9gBaebbnrfifHhDYfgasaacH8akY=wiFfYdH8Gipec8Eeeu0xXdbba9frFj0=OqFfea0dXdd9vqai=hGuQ8kuc9pgc9s8qqaq=dirpe0xb9q8qiLsFr0=vr0=vr0dc8meaabaqaciaacaGaaeqabaqabeGadaaakeaacqWGnbqtdaqhaaWcbaGaemyAaKMaeiilaWIaemOAaOgabaGaeGimaadaaaaa@3282@(*A*) = {(0, 0)}. We first consider the case where **a**_*i *_and **b**_*j *_are aligned. For each (*g*, *f*) in Sai
 MathType@MTEF@5@5@+=feaafiart1ev1aaatCvAUfKttLearuWrP9MDH5MBPbIqV92AaeXatLxBI9gBaebbnrfifHhDYfgasaacH8akY=wiFfYdH8Gipec8Eeeu0xXdbba9frFj0=OqFfea0dXdd9vqai=hGuQ8kuc9pgc9s8qqaq=dirpe0xb9q8qiLsFr0=vr0=vr0dc8meaabaqaciaacaGaaeqabaqabeGadaaakeaacqWGtbWudaWgaaWcbaacbeGae8xyae2aaSbaaWqaaiabdMgaPbqabaaaleqaaaaa@30EB@, if there exists element (*s*, *d*) in Mi−1,j−10
 MathType@MTEF@5@5@+=feaafiart1ev1aaatCvAUfKttLearuWrP9MDH5MBPbIqV92AaeXatLxBI9gBaebbnrfifHhDYfgasaacH8akY=wiFfYdH8Gipec8Eeeu0xXdbba9frFj0=OqFfea0dXdd9vqai=hGuQ8kuc9pgc9s8qqaq=dirpe0xb9q8qiLsFr0=vr0=vr0dc8meaabaqaciaacaGaaeqabaqabeGadaaakeaacqWGnbqtdaqhaaWcbaGaemyAaKMaeyOeI0IaeGymaeJaeiilaWIaemOAaOMaeyOeI0IaeGymaedabaGaeGimaadaaaaa@363C@(*A*) such that *g *= *s*, then (*s *+ 1, *d*) ∈ Mi,j0
 MathType@MTEF@5@5@+=feaafiart1ev1aaatCvAUfKttLearuWrP9MDH5MBPbIqV92AaeXatLxBI9gBaebbnrfifHhDYfgasaacH8akY=wiFfYdH8Gipec8Eeeu0xXdbba9frFj0=OqFfea0dXdd9vqai=hGuQ8kuc9pgc9s8qqaq=dirpe0xb9q8qiLsFr0=vr0=vr0dc8meaabaqaciaacaGaaeqabaqabeGadaaakeaacqWGnbqtdaqhaaWcbaGaemyAaKMaeiilaWIaemOAaOgabaGaeGimaadaaaaa@3282@(*A*). In the case where **b**_*j *_is aligned with a null column, then (*s*, *d *+ 1) ∈ Mi,j0
 MathType@MTEF@5@5@+=feaafiart1ev1aaatCvAUfKttLearuWrP9MDH5MBPbIqV92AaeXatLxBI9gBaebbnrfifHhDYfgasaacH8akY=wiFfYdH8Gipec8Eeeu0xXdbba9frFj0=OqFfea0dXdd9vqai=hGuQ8kuc9pgc9s8qqaq=dirpe0xb9q8qiLsFr0=vr0=vr0dc8meaabaqaciaacaGaaeqabaqabeGadaaakeaacqWGnbqtdaqhaaWcbaGaemyAaKMaeiilaWIaemOAaOgabaGaeGimaadaaaaa@3282@(*A*) where (*s*, *d*) is an element in Mi,j−10
 MathType@MTEF@5@5@+=feaafiart1ev1aaatCvAUfKttLearuWrP9MDH5MBPbIqV92AaeXatLxBI9gBaebbnrfifHhDYfgasaacH8akY=wiFfYdH8Gipec8Eeeu0xXdbba9frFj0=OqFfea0dXdd9vqai=hGuQ8kuc9pgc9s8qqaq=dirpe0xb9q8qiLsFr0=vr0=vr0dc8meaabaqaciaacaGaaeqabaqabeGadaaakeaacqWGnbqtdaqhaaWcbaGaemyAaKMaeiilaWIaemOAaOMaeyOeI0IaeGymaedabaGaeGimaadaaaaa@345F@(*A*) or Mi,j−13
 MathType@MTEF@5@5@+=feaafiart1ev1aaatCvAUfKttLearuWrP9MDH5MBPbIqV92AaeXatLxBI9gBaebbnrfifHhDYfgasaacH8akY=wiFfYdH8Gipec8Eeeu0xXdbba9frFj0=OqFfea0dXdd9vqai=hGuQ8kuc9pgc9s8qqaq=dirpe0xb9q8qiLsFr0=vr0=vr0dc8meaabaqaciaacaGaaeqabaqabeGadaaakeaacqWGnbqtdaqhaaWcbaGaemyAaKMaeiilaWIaemOAaOMaeyOeI0IaeGymaedabaGaeG4mamdaaaaa@3465@(*A*) depending on the maximum operation of equation 4. If **a**_*i *_is aligned with a null column, Mi,j0
 MathType@MTEF@5@5@+=feaafiart1ev1aaatCvAUfKttLearuWrP9MDH5MBPbIqV92AaeXatLxBI9gBaebbnrfifHhDYfgasaacH8akY=wiFfYdH8Gipec8Eeeu0xXdbba9frFj0=OqFfea0dXdd9vqai=hGuQ8kuc9pgc9s8qqaq=dirpe0xb9q8qiLsFr0=vr0=vr0dc8meaabaqaciaacaGaaeqabaqabeGadaaakeaacqWGnbqtdaqhaaWcbaGaemyAaKMaeiilaWIaemOAaOgabaGaeGimaadaaaaa@3282@(*A*) equals Mi−1,j0
 MathType@MTEF@5@5@+=feaafiart1ev1aaatCvAUfKttLearuWrP9MDH5MBPbIqV92AaeXatLxBI9gBaebbnrfifHhDYfgasaacH8akY=wiFfYdH8Gipec8Eeeu0xXdbba9frFj0=OqFfea0dXdd9vqai=hGuQ8kuc9pgc9s8qqaq=dirpe0xb9q8qiLsFr0=vr0=vr0dc8meaabaqaciaacaGaaeqabaqabeGadaaakeaacqWGnbqtdaqhaaWcbaGaemyAaKMaeyOeI0IaeGymaeJaeiilaWIaemOAaOgabaGaeGimaadaaaaa@345F@(*A*) or Mi−1,j2
 MathType@MTEF@5@5@+=feaafiart1ev1aaatCvAUfKttLearuWrP9MDH5MBPbIqV92AaeXatLxBI9gBaebbnrfifHhDYfgasaacH8akY=wiFfYdH8Gipec8Eeeu0xXdbba9frFj0=OqFfea0dXdd9vqai=hGuQ8kuc9pgc9s8qqaq=dirpe0xb9q8qiLsFr0=vr0=vr0dc8meaabaqaciaacaGaaeqabaqabeGadaaakeaacqWGnbqtdaqhaaWcbaGaemyAaKMaeyOeI0IaeGymaeJaeiilaWIaemOAaOgabaGaeGOmaidaaaaa@3463@(*A*). In the case of Figure 1, M7,110
 MathType@MTEF@5@5@+=feaafiart1ev1aaatCvAUfKttLearuWrP9MDH5MBPbIqV92AaeXatLxBI9gBaebbnrfifHhDYfgasaacH8akY=wiFfYdH8Gipec8Eeeu0xXdbba9frFj0=OqFfea0dXdd9vqai=hGuQ8kuc9pgc9s8qqaq=dirpe0xb9q8qiLsFr0=vr0=vr0dc8meaabaqaciaacaGaaeqabaqabeGadaaakeaacqWGnbqtdaqhaaWcbaGaeG4naCJaeiilaWIaeGymaeJaeGymaedabaGaeGimaadaaaaa@32A6@(*A*) = {(0, 1), (2, 1), (7, 4)} is derived from M7,100
 MathType@MTEF@5@5@+=feaafiart1ev1aaatCvAUfKttLearuWrP9MDH5MBPbIqV92AaeXatLxBI9gBaebbnrfifHhDYfgasaacH8akY=wiFfYdH8Gipec8Eeeu0xXdbba9frFj0=OqFfea0dXdd9vqai=hGuQ8kuc9pgc9s8qqaq=dirpe0xb9q8qiLsFr0=vr0=vr0dc8meaabaqaciaacaGaaeqabaqabeGadaaakeaacqWGnbqtdaqhaaWcbaGaeG4naCJaeiilaWIaeGymaeJaeGimaadabaGaeGimaadaaaaa@32A4@(*A*) = {(0, 0), (2, 0), (7, 3)}. The other gap mediation profile vectors are constructed in a similar way.

Running gap profile vectors E^ai+
 MathType@MTEF@5@5@+=feaafiart1ev1aaatCvAUfKttLearuWrP9MDH5MBPbIqV92AaeXatLxBI9gBaebbnrfifHhDYfgasaacH8akY=wiFfYdH8Gipec8Eeeu0xXdbba9frFj0=OqFfea0dXdd9vqai=hGuQ8kuc9pgc9s8qqaq=dirpe0xb9q8qiLsFr0=vr0=vr0dc8meaabaqaciaacaGaaeqabaqabeGadaaakeaacuWGfbqrgaqcamaaDaaaleaaieqacqWFHbqydaWgaaadbaGaemyAaKgabeaaaSqaaiabgUcaRaaaaaa@31C2@ and S^ai
 MathType@MTEF@5@5@+=feaafiart1ev1aaatCvAUfKttLearuWrP9MDH5MBPbIqV92AaeXatLxBI9gBaebbnrfifHhDYfgasaacH8akY=wiFfYdH8Gipec8Eeeu0xXdbba9frFj0=OqFfea0dXdd9vqai=hGuQ8kuc9pgc9s8qqaq=dirpe0xb9q8qiLsFr0=vr0=vr0dc8meaabaqaciaacaGaaeqabaqabeGadaaakeaacuWGtbWugaqcamaaBaaaleaaieqacqWFHbqydaWgaaadbaGaemyAaKgabeaaaSqabaaaaa@30FB@ are obtained by combining gap mediation profile Mi−1,j−10
 MathType@MTEF@5@5@+=feaafiart1ev1aaatCvAUfKttLearuWrP9MDH5MBPbIqV92AaeXatLxBI9gBaebbnrfifHhDYfgasaacH8akY=wiFfYdH8Gipec8Eeeu0xXdbba9frFj0=OqFfea0dXdd9vqai=hGuQ8kuc9pgc9s8qqaq=dirpe0xb9q8qiLsFr0=vr0=vr0dc8meaabaqaciaacaGaaeqabaqabeGadaaakeaacqWGnbqtdaqhaaWcbaGaemyAaKMaeyOeI0IaeGymaeJaeiilaWIaemOAaOMaeyOeI0IaeGymaedabaGaeGimaadaaaaa@363C@(*A*), and static gap profiles Eai+
 MathType@MTEF@5@5@+=feaafiart1ev1aaatCvAUfKttLearuWrP9MDH5MBPbIqV92AaeXatLxBI9gBaebbnrfifHhDYfgasaacH8akY=wiFfYdH8Gipec8Eeeu0xXdbba9frFj0=OqFfea0dXdd9vqai=hGuQ8kuc9pgc9s8qqaq=dirpe0xb9q8qiLsFr0=vr0=vr0dc8meaabaqaciaacaGaaeqabaqabeGadaaakeaacqWGfbqrdaqhaaWcbaacbeGae8xyae2aaSbaaWqaaiabdMgaPbqabaaaleaacqGHRaWkaaaaaa@31B2@ and Sai
 MathType@MTEF@5@5@+=feaafiart1ev1aaatCvAUfKttLearuWrP9MDH5MBPbIqV92AaeXatLxBI9gBaebbnrfifHhDYfgasaacH8akY=wiFfYdH8Gipec8Eeeu0xXdbba9frFj0=OqFfea0dXdd9vqai=hGuQ8kuc9pgc9s8qqaq=dirpe0xb9q8qiLsFr0=vr0=vr0dc8meaabaqaciaacaGaaeqabaqabeGadaaakeaacqWGtbWudaWgaaWcbaacbeGae8xyae2aaSbaaWqaaiabdMgaPbqabaaaleqaaaaa@30EB@, respectively. For each (*g*, *f*^+^) in Eai+
 MathType@MTEF@5@5@+=feaafiart1ev1aaatCvAUfKttLearuWrP9MDH5MBPbIqV92AaeXatLxBI9gBaebbnrfifHhDYfgasaacH8akY=wiFfYdH8Gipec8Eeeu0xXdbba9frFj0=OqFfea0dXdd9vqai=hGuQ8kuc9pgc9s8qqaq=dirpe0xb9q8qiLsFr0=vr0=vr0dc8meaabaqaciaacaGaaeqabaqabeGadaaakeaacqWGfbqrdaqhaaWcbaacbeGae8xyae2aaSbaaWqaaiabdMgaPbqabaaaleaacqGHRaWkaaaaaa@31B2@, (*s*, *d*) is chosen from Mi−1,j−10
 MathType@MTEF@5@5@+=feaafiart1ev1aaatCvAUfKttLearuWrP9MDH5MBPbIqV92AaeXatLxBI9gBaebbnrfifHhDYfgasaacH8akY=wiFfYdH8Gipec8Eeeu0xXdbba9frFj0=OqFfea0dXdd9vqai=hGuQ8kuc9pgc9s8qqaq=dirpe0xb9q8qiLsFr0=vr0=vr0dc8meaabaqaciaacaGaaeqabaqabeGadaaakeaacqWGnbqtdaqhaaWcbaGaemyAaKMaeyOeI0IaeGymaeJaeiilaWIaemOAaOMaeyOeI0IaeGymaedabaGaeGimaadaaaaa@363C@(*A*) such that *g *= *s*, and then (*s *+ *d*, *f*^+^) ∈ E^ai+
 MathType@MTEF@5@5@+=feaafiart1ev1aaatCvAUfKttLearuWrP9MDH5MBPbIqV92AaeXatLxBI9gBaebbnrfifHhDYfgasaacH8akY=wiFfYdH8Gipec8Eeeu0xXdbba9frFj0=OqFfea0dXdd9vqai=hGuQ8kuc9pgc9s8qqaq=dirpe0xb9q8qiLsFr0=vr0=vr0dc8meaabaqaciaacaGaaeqabaqabeGadaaakeaacuWGfbqrgaqcamaaDaaaleaaieqacqWFHbqydaWgaaadbaGaemyAaKgabeaaaSqaaiabgUcaRaaaaaa@31C2@. Similarly, S^ai
 MathType@MTEF@5@5@+=feaafiart1ev1aaatCvAUfKttLearuWrP9MDH5MBPbIqV92AaeXatLxBI9gBaebbnrfifHhDYfgasaacH8akY=wiFfYdH8Gipec8Eeeu0xXdbba9frFj0=OqFfea0dXdd9vqai=hGuQ8kuc9pgc9s8qqaq=dirpe0xb9q8qiLsFr0=vr0=vr0dc8meaabaqaciaacaGaaeqabaqabeGadaaakeaacuWGtbWugaqcamaaBaaaleaaieqacqWFHbqydaWgaaadbaGaemyAaKgabeaaaSqabaaaaa@30FB@ is obtained from Mi−1,j−10
 MathType@MTEF@5@5@+=feaafiart1ev1aaatCvAUfKttLearuWrP9MDH5MBPbIqV92AaeXatLxBI9gBaebbnrfifHhDYfgasaacH8akY=wiFfYdH8Gipec8Eeeu0xXdbba9frFj0=OqFfea0dXdd9vqai=hGuQ8kuc9pgc9s8qqaq=dirpe0xb9q8qiLsFr0=vr0=vr0dc8meaabaqaciaacaGaaeqabaqabeGadaaakeaacqWGnbqtdaqhaaWcbaGaemyAaKMaeyOeI0IaeGymaeJaeiilaWIaemOAaOMaeyOeI0IaeGymaedabaGaeGimaadaaaaa@363C@(*A*) and Sai
 MathType@MTEF@5@5@+=feaafiart1ev1aaatCvAUfKttLearuWrP9MDH5MBPbIqV92AaeXatLxBI9gBaebbnrfifHhDYfgasaacH8akY=wiFfYdH8Gipec8Eeeu0xXdbba9frFj0=OqFfea0dXdd9vqai=hGuQ8kuc9pgc9s8qqaq=dirpe0xb9q8qiLsFr0=vr0=vr0dc8meaabaqaciaacaGaaeqabaqabeGadaaakeaacqWGtbWudaWgaaWcbaacbeGae8xyae2aaSbaaWqaaiabdMgaPbqabaaaleqaaaaa@30EB@. For example, E^a8+
 MathType@MTEF@5@5@+=feaafiart1ev1aaatCvAUfKttLearuWrP9MDH5MBPbIqV92AaeXatLxBI9gBaebbnrfifHhDYfgasaacH8akY=wiFfYdH8Gipec8Eeeu0xXdbba9frFj0=OqFfea0dXdd9vqai=hGuQ8kuc9pgc9s8qqaq=dirpe0xb9q8qiLsFr0=vr0=vr0dc8meaabaqaciaacaGaaeqabaqabeGadaaakeaacuWGfbqrgaqcamaaDaaaleaaieqacqWFHbqydaWgaaadbaGaeGioaGdabeaaaSqaaiabgUcaRaaaaaa@3165@ and S^a8
 MathType@MTEF@5@5@+=feaafiart1ev1aaatCvAUfKttLearuWrP9MDH5MBPbIqV92AaeXatLxBI9gBaebbnrfifHhDYfgasaacH8akY=wiFfYdH8Gipec8Eeeu0xXdbba9frFj0=OqFfea0dXdd9vqai=hGuQ8kuc9pgc9s8qqaq=dirpe0xb9q8qiLsFr0=vr0=vr0dc8meaabaqaciaacaGaaeqabaqabeGadaaakeaacuWGtbWugaqcamaaBaaaleaaieqacqWFHbqydaWgaaadbaGaeGioaGdabeaaaSqabaaaaa@309E@ at the node (8, 12) of Figure 1 are {(1, wA2
 MathType@MTEF@5@5@+=feaafiart1ev1aaatCvAUfKttLearuWrP9MDH5MBPbIqV92AaeXatLxBI9gBaebbnrfifHhDYfgasaacH8akY=wiFfYdH8Gipec8Eeeu0xXdbba9frFj0=OqFfea0dXdd9vqai=hGuQ8kuc9pgc9s8qqaq=dirpe0xb9q8qiLsFr0=vr0=vr0dc8meaabaqaciaacaGaaeqabaqabeGadaaakeaacqWG3bWDdaWgaaWcbaGaemyqae0aaSbaaWqaaiabikdaYaqabaaaleqaaaaa@3084@ + wA3
 MathType@MTEF@5@5@+=feaafiart1ev1aaatCvAUfKttLearuWrP9MDH5MBPbIqV92AaeXatLxBI9gBaebbnrfifHhDYfgasaacH8akY=wiFfYdH8Gipec8Eeeu0xXdbba9frFj0=OqFfea0dXdd9vqai=hGuQ8kuc9pgc9s8qqaq=dirpe0xb9q8qiLsFr0=vr0=vr0dc8meaabaqaciaacaGaaeqabaqabeGadaaakeaacqWG3bWDdaWgaaWcbaGaemyqae0aaSbaaWqaaiabiodaZaqabaaaleqaaaaa@3086@ + wA4
 MathType@MTEF@5@5@+=feaafiart1ev1aaatCvAUfKttLearuWrP9MDH5MBPbIqV92AaeXatLxBI9gBaebbnrfifHhDYfgasaacH8akY=wiFfYdH8Gipec8Eeeu0xXdbba9frFj0=OqFfea0dXdd9vqai=hGuQ8kuc9pgc9s8qqaq=dirpe0xb9q8qiLsFr0=vr0=vr0dc8meaabaqaciaacaGaaeqabaqabeGadaaakeaacqWG3bWDdaWgaaWcbaGaemyqae0aaSbaaWqaaiabisda0aqabaaaleqaaaaa@3088@), (3, wA2
 MathType@MTEF@5@5@+=feaafiart1ev1aaatCvAUfKttLearuWrP9MDH5MBPbIqV92AaeXatLxBI9gBaebbnrfifHhDYfgasaacH8akY=wiFfYdH8Gipec8Eeeu0xXdbba9frFj0=OqFfea0dXdd9vqai=hGuQ8kuc9pgc9s8qqaq=dirpe0xb9q8qiLsFr0=vr0=vr0dc8meaabaqaciaacaGaaeqabaqabeGadaaakeaacqWG3bWDdaWgaaWcbaGaemyqae0aaSbaaWqaaiabikdaYaqabaaaleqaaaaa@3084@ + wA3
 MathType@MTEF@5@5@+=feaafiart1ev1aaatCvAUfKttLearuWrP9MDH5MBPbIqV92AaeXatLxBI9gBaebbnrfifHhDYfgasaacH8akY=wiFfYdH8Gipec8Eeeu0xXdbba9frFj0=OqFfea0dXdd9vqai=hGuQ8kuc9pgc9s8qqaq=dirpe0xb9q8qiLsFr0=vr0=vr0dc8meaabaqaciaacaGaaeqabaqabeGadaaakeaacqWG3bWDdaWgaaWcbaGaemyqae0aaSbaaWqaaiabiodaZaqabaaaleqaaaaa@3086@), (11, wA2
 MathType@MTEF@5@5@+=feaafiart1ev1aaatCvAUfKttLearuWrP9MDH5MBPbIqV92AaeXatLxBI9gBaebbnrfifHhDYfgasaacH8akY=wiFfYdH8Gipec8Eeeu0xXdbba9frFj0=OqFfea0dXdd9vqai=hGuQ8kuc9pgc9s8qqaq=dirpe0xb9q8qiLsFr0=vr0=vr0dc8meaabaqaciaacaGaaeqabaqabeGadaaakeaacqWG3bWDdaWgaaWcbaGaemyqae0aaSbaaWqaaiabikdaYaqabaaaleqaaaaa@3084@)} and {(1, wA1
 MathType@MTEF@5@5@+=feaafiart1ev1aaatCvAUfKttLearuWrP9MDH5MBPbIqV92AaeXatLxBI9gBaebbnrfifHhDYfgasaacH8akY=wiFfYdH8Gipec8Eeeu0xXdbba9frFj0=OqFfea0dXdd9vqai=hGuQ8kuc9pgc9s8qqaq=dirpe0xb9q8qiLsFr0=vr0=vr0dc8meaabaqaciaacaGaaeqabaqabeGadaaakeaacqWG3bWDdaWgaaWcbaGaemyqae0aaSbaaWqaaiabigdaXaqabaaaleqaaaaa@3082@)}, respectively.

Using running gap profile vectors E^ai+
 MathType@MTEF@5@5@+=feaafiart1ev1aaatCvAUfKttLearuWrP9MDH5MBPbIqV92AaeXatLxBI9gBaebbnrfifHhDYfgasaacH8akY=wiFfYdH8Gipec8Eeeu0xXdbba9frFj0=OqFfea0dXdd9vqai=hGuQ8kuc9pgc9s8qqaq=dirpe0xb9q8qiLsFr0=vr0=vr0dc8meaabaqaciaacaGaaeqabaqabeGadaaakeaacuWGfbqrgaqcamaaDaaaleaaieqacqWFHbqydaWgaaadbaGaemyAaKgabeaaaSqaaiabgUcaRaaaaaa@31C2@, S^ai
 MathType@MTEF@5@5@+=feaafiart1ev1aaatCvAUfKttLearuWrP9MDH5MBPbIqV92AaeXatLxBI9gBaebbnrfifHhDYfgasaacH8akY=wiFfYdH8Gipec8Eeeu0xXdbba9frFj0=OqFfea0dXdd9vqai=hGuQ8kuc9pgc9s8qqaq=dirpe0xb9q8qiLsFr0=vr0=vr0dc8meaabaqaciaacaGaaeqabaqabeGadaaakeaacuWGtbWugaqcamaaBaaaleaaieqacqWFHbqydaWgaaadbaGaemyAaKgabeaaaSqabaaaaa@30FB@, E^bj+
 MathType@MTEF@5@5@+=feaafiart1ev1aaatCvAUfKttLearuWrP9MDH5MBPbIqV92AaeXatLxBI9gBaebbnrfifHhDYfgasaacH8akY=wiFfYdH8Gipec8Eeeu0xXdbba9frFj0=OqFfea0dXdd9vqai=hGuQ8kuc9pgc9s8qqaq=dirpe0xb9q8qiLsFr0=vr0=vr0dc8meaabaqaciaacaGaaeqabaqabeGadaaakeaacuWGfbqrgaqcamaaDaaaleaaieqacqWFIbGydaWgaaadbaGaemOAaOgabeaaaSqaaiabgUcaRaaaaaa@31C6@, and S^bj
 MathType@MTEF@5@5@+=feaafiart1ev1aaatCvAUfKttLearuWrP9MDH5MBPbIqV92AaeXatLxBI9gBaebbnrfifHhDYfgasaacH8akY=wiFfYdH8Gipec8Eeeu0xXdbba9frFj0=OqFfea0dXdd9vqai=hGuQ8kuc9pgc9s8qqaq=dirpe0xb9q8qiLsFr0=vr0=vr0dc8meaabaqaciaacaGaaeqabaqabeGadaaakeaacuWGtbWugaqcamaaBaaaleaaieqacqWFIbGydaWgaaadbaGaemOAaOgabeaaaSqabaaaaa@30FF@, Equation 6 can be rewritten as:

G(ai,bj;Pi−1,j−10)=(−v)⋅(S^bj·E^ai++S^ai·E^bj+).     (8)
 MathType@MTEF@5@5@+=feaafiart1ev1aaatCvAUfKttLearuWrP9MDH5MBPbIqV92AaeXatLxBI9gBaebbnrfifHhDYfgasaacH8akY=wiFfYdH8Gipec8Eeeu0xXdbba9frFj0=OqFfea0dXdd9vqai=hGuQ8kuc9pgc9s8qqaq=dirpe0xb9q8qiLsFr0=vr0=vr0dc8meaabaqaciaacaGaaeqabaqabeGadaaakeaacqWGhbWrcqGGOaakieqacqWFHbqydaWgaaWcbaGaemyAaKgabeaakiabcYcaSiab=jgaInaaBaaaleaacqWGQbGAaeqaaOGaei4oaSJaemiuaa1aa0baaSqaaiabdMgaPjabgkHiTiabigdaXiabcYcaSiabdQgaQjabgkHiTiabigdaXaqaaiabicdaWaaakiabcMcaPiabg2da9iabcIcaOiabgkHiTiabdAha2jabcMcaPiabgwSixlabcIcaOiqbdofatzaajaWaaSbaaSqaaiab=jgaInaaBaaameaacqWGQbGAaeqaaaWcbeaakiabl+y6NjqbdweafzaajaWaa0baaSqaaiab=fgaHnaaBaaameaacqWGPbqAaeqaaaWcbaGaey4kaScaaOGaey4kaSIafm4uamLbaKaadaWgaaWcbaGae8xyae2aaSbaaWqaaiabdMgaPbqabaaaleqaaOGaeS4JPFMafmyrauKbaKaadaqhaaWcbaGae8Nyai2aaSbaaWqaaiabdQgaQbqabaaaleaacqGHRaWkaaGccqGGPaqkcqGGUaGlcaWLjaGaaCzcamaabmaabaGaeGioaGdacaGLOaGaayzkaaaaaa@670A@

*S *• *E*^+ ^is expressed as

S·E+=∑1≤p≤|S|f(sp)⋅f(eq+)
 MathType@MTEF@5@5@+=feaafiart1ev1aaatCvAUfKttLearuWrP9MDH5MBPbIqV92AaeXatLxBI9gBaebbnrfifHhDYfgasaacH8akY=wiFfYdH8Gipec8Eeeu0xXdbba9frFj0=OqFfea0dXdd9vqai=hGuQ8kuc9pgc9s8qqaq=dirpe0xb9q8qiLsFr0=vr0=vr0dc8meaabaqaciaacaGaaeqabaqabeGadaaakeaacqWGtbWucqWIpM+zcqWGfbqrdaahaaWcbeqaaiabgUcaRaaakiabg2da9maaqafabaGaemOzayMaeiikaGIaem4Cam3aaSbaaSqaaiabdchaWbqabaGccqGGPaqkcqGHflY1cqWGMbGzcqGGOaakcqWGLbqzdaqhaaWcbaGaemyCaehabaGaey4kaScaaOGaeiykaKcaleaacqaIXaqmcqGHKjYOcqWGWbaCcqGHKjYOcqGG8baFcqWGtbWucqGG8baFaeqaniabggHiLdaaaa@4ECF@

where *s*_*p *_and eq+
 MathType@MTEF@5@5@+=feaafiart1ev1aaatCvAUfKttLearuWrP9MDH5MBPbIqV92AaeXatLxBI9gBaebbnrfifHhDYfgasaacH8akY=wiFfYdH8Gipec8Eeeu0xXdbba9frFj0=OqFfea0dXdd9vqai=hGuQ8kuc9pgc9s8qqaq=dirpe0xb9q8qiLsFr0=vr0=vr0dc8meaabaqaciaacaGaaeqabaqabeGadaaakeaacqWGLbqzdaqhaaWcbaGaemyCaehabaGaey4kaScaaaaa@3079@ are the *p*-th and *q*-th elements in *S *and *E*^+^, respectively, and *f*(*e*) is the weighted frequency of element *e*. *q *is chosen such that the gap state of eq+
 MathType@MTEF@5@5@+=feaafiart1ev1aaatCvAUfKttLearuWrP9MDH5MBPbIqV92AaeXatLxBI9gBaebbnrfifHhDYfgasaacH8akY=wiFfYdH8Gipec8Eeeu0xXdbba9frFj0=OqFfea0dXdd9vqai=hGuQ8kuc9pgc9s8qqaq=dirpe0xb9q8qiLsFr0=vr0=vr0dc8meaabaqaciaacaGaaeqabaqabeGadaaakeaacqWGLbqzdaqhaaWcbaGaemyCaehabaGaey4kaScaaaaa@3079@ is the smallest among the elements in *E*^+ ^whose gap states are not less than that of *s*_*k*_. Calculation of *S *• *E*^+ ^requires *O*(|*S*|) + |*E*^+^|), and hence *G*(**a**_*i*_, **b**_*j*_; Pi−1,j−10
 MathType@MTEF@5@5@+=feaafiart1ev1aaatCvAUfKttLearuWrP9MDH5MBPbIqV92AaeXatLxBI9gBaebbnrfifHhDYfgasaacH8akY=wiFfYdH8Gipec8Eeeu0xXdbba9frFj0=OqFfea0dXdd9vqai=hGuQ8kuc9pgc9s8qqaq=dirpe0xb9q8qiLsFr0=vr0=vr0dc8meaabaqaciaacaGaaeqabaqabeGadaaakeaacqWGqbaudaqhaaWcbaGaemyAaKMaeyOeI0IaeGymaeJaeiilaWIaemOAaOMaeyOeI0IaeGymaedabaGaeGimaadaaaaa@3642@) at most *O*(*m *+ *n*). Using equation 8 instead of equation 6 can reduce computation from *O*(*mn*) to *O*(*m *+ *n*) even in the worst case where gap states on a column are mutually different.

### Novel group-to-group sequence alignment algorithm with piecewise linear gap cost

In this section, we describe a novel group-to-group sequence alignment algorithm with a piecewise linear gap cost. Although this algorithm uses recurrent equations 1 to 4, the algorithms calculating *S*(**a**_*i*_, **b**_*j*_) and *G*(**a**_*i*_, **b**_*j*_; Pi−1,j−10
 MathType@MTEF@5@5@+=feaafiart1ev1aaatCvAUfKttLearuWrP9MDH5MBPbIqV92AaeXatLxBI9gBaebbnrfifHhDYfgasaacH8akY=wiFfYdH8Gipec8Eeeu0xXdbba9frFj0=OqFfea0dXdd9vqai=hGuQ8kuc9pgc9s8qqaq=dirpe0xb9q8qiLsFr0=vr0=vr0dc8meaabaqaciaacaGaaeqabaqabeGadaaakeaacqWGqbaudaqhaaWcbaGaemyAaKMaeyOeI0IaeGymaeJaeiilaWIaemOAaOMaeyOeI0IaeGymaedabaGaeGimaadaaaaa@3642@) must be changed. Roughly speaking, the term for calculating gap extension penalties is transferred from *S*(**a**_*i*_, **b**_*j*_) to *G*(**a**_*i*_, **b**_*j*_; Pi−1,j−10
 MathType@MTEF@5@5@+=feaafiart1ev1aaatCvAUfKttLearuWrP9MDH5MBPbIqV92AaeXatLxBI9gBaebbnrfifHhDYfgasaacH8akY=wiFfYdH8Gipec8Eeeu0xXdbba9frFj0=OqFfea0dXdd9vqai=hGuQ8kuc9pgc9s8qqaq=dirpe0xb9q8qiLsFr0=vr0=vr0dc8meaabaqaciaacaGaaeqabaqabeGadaaakeaacqWGqbaudaqhaaWcbaGaemyAaKMaeyOeI0IaeGymaeJaeiilaWIaemOAaOMaeyOeI0IaeGymaedabaGaeGimaadaaaaa@3642@). After explanation of the piecewise linear gap cost, we describe these algorithms in detail.

#### Piecewise linear gap cost

The piecewise linear gap cost consists of several linear functions [18]:

g(x)=max⁡1≤l≤L{−(ulx+vl)}     (9)
 MathType@MTEF@5@5@+=feaafiart1ev1aaatCvAUfKttLearuWrP9MDH5MBPbIqV92AaeXatLxBI9gBaebbnrfifHhDYfgasaacH8akY=wiFfYdH8Gipec8Eeeu0xXdbba9frFj0=OqFfea0dXdd9vqai=hGuQ8kuc9pgc9s8qqaq=dirpe0xb9q8qiLsFr0=vr0=vr0dc8meaabaqaciaacaGaaeqabaqabeGadaaakeaacqWGNbWzcqGGOaakcqWG4baEcqGGPaqkcqGH9aqpdaWfqaqaaiGbc2gaTjabcggaHjabcIha4bWcbaGaeGymaeJaeyizImQaemiBaWMaeyizImQaemitaWeabeaakiabcUha7jabgkHiTiabcIcaOiabdwha1naaBaaaleaacqWGSbaBaeqaaOGaemiEaGNaey4kaSIaemODay3aaSbaaSqaaiabdYgaSbqabaGccqGGPaqkcqGG9bqFcaWLjaGaaCzcamaabmaabaGaeGyoaKdacaGLOaGaayzkaaaaaa@4F56@

where *u*_*l *_> *u*_*l*+1_(≥ 0) and *v*_*l*+1 _> *v*_*l*_(> 0). When *L *= 1, this cost is the same as the affine gap cost. This cost could alleviate over-penalizing long indels, because the inclination of *g*(*x*), which corresponds to a gap extension penalty, *u*_*l*_, becomes small as gap length increases. In other words, this cost calculates gap extension penalties based on gap length. For the sake of simplicity, we restricted our attention to the case of *L *= 2. Then, *g*(*x*) = -(*u*_1_*x *+ *v*_1_) if *x *≤ *x*_*c *_or *g*(*x*) = -(*u*_2_*x *+ *v*_2_), otherwise, *x*_*c *_= ⌊(*v*_2 _- *v*_1_)/(*u*_1 _- *u*_2_)⌋ is called the critical gap length.

#### Calculation of substitution score

As mentioned above, the calculation of gap extension penalties must be separated from the calculation of the substitution score *S*(**a**_*i*_, **b**_*j*_) in order to use the piecewise linear gap cost. Therefore, *S*(**a**_*i*_, **b**_*j*_) is expressed as:

S(ai,bj)=∑r∈Σfai,r⋅pbj,r=∑r∈Σpai,r⋅fbj,r.     (10)
 MathType@MTEF@5@5@+=feaafiart1ev1aaatCvAUfKttLearuWrP9MDH5MBPbIqV92AaeXatLxBI9gBaebbnrfifHhDYfgasaacH8akY=wiFfYdH8Gipec8Eeeu0xXdbba9frFj0=OqFfea0dXdd9vqai=hGuQ8kuc9pgc9s8qqaq=dirpe0xb9q8qiLsFr0=vr0=vr0dc8meaabaqaciaacaGaaeqabaqabeGadaaakeaacqWGtbWucqGGOaakieqacqWFHbqydaWgaaWcbaGaemyAaKgabeaakiabcYcaSiab=jgaInaaBaaaleaacqWGQbGAaeqaaOGaeiykaKIaeyypa0ZaaabuaeaacqWGMbGzdaWgaaWcbaGae8xyae2aaSbaaWqaaiabdMgaPbqabaWccqGGSaalcqWGYbGCaeqaaOGaeyyXICTaemiCaa3aaSbaaSqaaiab=jgaInaaBaaameaacqWGQbGAaeqaaSGaeiilaWIaemOCaihabeaaaeaacqWGYbGCcqGHiiIZcqqHJoWuaeqaniabggHiLdGccqGH9aqpdaaeqbqaaiabdchaWnaaBaaaleaacqWFHbqydaWgaaadbaGaemyAaKgabeaaliabcYcaSiabdkhaYbqabaGccqGHflY1cqWGMbGzdaWgaaWcbaGae8Nyai2aaSbaaWqaaiabdQgaQbqabaWccqGGSaalcqWGYbGCaeqaaaqaaiabdkhaYjabgIGiolabfo6atbqab0GaeyyeIuoakiabc6caUiaaxMaacaWLjaWaaeWaaeaacqaIXaqmcqaIWaamaiaawIcacaGLPaaaaaa@6A6C@

where pbj,r=∑t∈Σfbj,r⋅s(r,t)
 MathType@MTEF@5@5@+=feaafiart1ev1aaatCvAUfKttLearuWrP9MDH5MBPbIqV92AaeXatLxBI9gBaebbnrfifHhDYfgasaacH8akY=wiFfYdH8Gipec8Eeeu0xXdbba9frFj0=OqFfea0dXdd9vqai=hGuQ8kuc9pgc9s8qqaq=dirpe0xb9q8qiLsFr0=vr0=vr0dc8meaabaqaciaacaGaaeqabaqabeGadaaakeaacqWGWbaCdaWgaaWcbaacbeGae8Nyai2aaSbaaWqaaiabdQgaQbqabaWccqGGSaalcqWGYbGCaeqaaOGaeyypa0ZaaabeaeaacqWGMbGzdaWgaaWcbaGae8Nyai2aaSbaaWqaaiabdQgaQbqabaWccqGGSaalcqWGYbGCaeqaaaqaaiabdsha0jabgIGiolabfo6atbqab0GaeyyeIuoakiabgwSixlabdohaZjabcIcaOiabdkhaYjabcYcaSiabdsha0jabcMcaPaaa@4AB6@. Note that this equation and the definition of residue profile vector sum over not Σ* but Σ.

#### Calculation of gap extension penalty

In the previous algorithm with an affine gap cost, *G*(**a**_*i*_, **b**_*j*_; Pi−1,j−10
 MathType@MTEF@5@5@+=feaafiart1ev1aaatCvAUfKttLearuWrP9MDH5MBPbIqV92AaeXatLxBI9gBaebbnrfifHhDYfgasaacH8akY=wiFfYdH8Gipec8Eeeu0xXdbba9frFj0=OqFfea0dXdd9vqai=hGuQ8kuc9pgc9s8qqaq=dirpe0xb9q8qiLsFr0=vr0=vr0dc8meaabaqaciaacaGaaeqabaqabeGadaaakeaacqWGqbaudaqhaaWcbaGaemyAaKMaeyOeI0IaeGymaeJaeiilaWIaemOAaOMaeyOeI0IaeGymaedabaGaeGimaadaaaaa@3642@) was responsible for the gap opening penalty only; however, it must take care of the gap extension penalty in the case of the piecewise linear gap cost. Therefore, a term for gap extension penalty is added to the right hand side of equation 8. Let us consider an example of gap extension penalty calculation for the null on **b**_12 _aligned with the residues on **a**_8 _(the last column of Figure 1). The gap state at *b*_1,12 _is 8. With omission of nulls that are aligned with other nulls, the lengths of the gap on *B*_1 _relative to *A*_2_, *A*_3_, and *A*_4 _are 1, 4, and 6, respectively. Assuming that the critical gap length *x*_*c *_= 4, we obtain the respective gap extension penalties for *A*_2_, *A*_3_, and *A*_4 _as *u*_1_, *u*_1_, and *u*_2_. Therefore, the gap extension penalty in question is wA1
 MathType@MTEF@5@5@+=feaafiart1ev1aaatCvAUfKttLearuWrP9MDH5MBPbIqV92AaeXatLxBI9gBaebbnrfifHhDYfgasaacH8akY=wiFfYdH8Gipec8Eeeu0xXdbba9frFj0=OqFfea0dXdd9vqai=hGuQ8kuc9pgc9s8qqaq=dirpe0xb9q8qiLsFr0=vr0=vr0dc8meaabaqaciaacaGaaeqabaqabeGadaaakeaacqWG3bWDdaWgaaWcbaGaemyqae0aaSbaaWqaaiabigdaXaqabaaaleqaaaaa@3082@ (*F*_1_·*u*_1 _+ *F*_2_·*u*_2_), where *F*_1 _= wA2
 MathType@MTEF@5@5@+=feaafiart1ev1aaatCvAUfKttLearuWrP9MDH5MBPbIqV92AaeXatLxBI9gBaebbnrfifHhDYfgasaacH8akY=wiFfYdH8Gipec8Eeeu0xXdbba9frFj0=OqFfea0dXdd9vqai=hGuQ8kuc9pgc9s8qqaq=dirpe0xb9q8qiLsFr0=vr0=vr0dc8meaabaqaciaacaGaaeqabaqabeGadaaakeaacqWG3bWDdaWgaaWcbaGaemyqae0aaSbaaWqaaiabikdaYaqabaaaleqaaaaa@3084@ + wA3
 MathType@MTEF@5@5@+=feaafiart1ev1aaatCvAUfKttLearuWrP9MDH5MBPbIqV92AaeXatLxBI9gBaebbnrfifHhDYfgasaacH8akY=wiFfYdH8Gipec8Eeeu0xXdbba9frFj0=OqFfea0dXdd9vqai=hGuQ8kuc9pgc9s8qqaq=dirpe0xb9q8qiLsFr0=vr0=vr0dc8meaabaqaciaacaGaaeqabaqabeGadaaakeaacqWG3bWDdaWgaaWcbaGaemyqae0aaSbaaWqaaiabiodaZaqabaaaleqaaaaa@3086@ and *F*_2 _= wA4
 MathType@MTEF@5@5@+=feaafiart1ev1aaatCvAUfKttLearuWrP9MDH5MBPbIqV92AaeXatLxBI9gBaebbnrfifHhDYfgasaacH8akY=wiFfYdH8Gipec8Eeeu0xXdbba9frFj0=OqFfea0dXdd9vqai=hGuQ8kuc9pgc9s8qqaq=dirpe0xb9q8qiLsFr0=vr0=vr0dc8meaabaqaciaacaGaaeqabaqabeGadaaakeaacqWG3bWDdaWgaaWcbaGaemyqae0aaSbaaWqaaiabisda0aqabaaaleqaaaaa@3088@.

This example indicates two important points for the exact calculation of gap extension penalty. First, each gap length is obtained by counting the residues on each row of a specific range in *A *called 'relevant segment', *A*(3, 8) in this example. Second, the two nulls on *B*_1 _at columns **b**_5 _and **b**_11 _are aligned with two separate dynamic gaps inserted into *A*. The first observation suggests that *F*_1 _and *F*_2 _for any segment may be calculated before the DP process. However, the second observation indicates that the number of null columns of dynamic gaps aligned with static nulls on a target gap must be subtracted from the gap state of the target gap for correct assignment of the relevant segment; without this subtraction, the relevant segment would be assigned as *A*(1, 8) in the above example.

We initially consider the first point, neglecting the presence of dynamic gaps for a moment. Without loss of generality, we assume that the residues on **a**_*i *_are aligned with a null of a target gap on **b**_*j *_whose gap state is *g*. We define *F*_0_(**a**_*i*_) as the weighted fraction of nulls on a_*i*_: F0(ai)≡∑p=1mwApδ(ap,i,−)
 MathType@MTEF@5@5@+=feaafiart1ev1aaatCvAUfKttLearuWrP9MDH5MBPbIqV92AaeXatLxBI9gBaebbnrfifHhDYfgasaacH8akY=wiFfYdH8Gipec8Eeeu0xXdbba9frFj0=OqFfea0dXdd9vqai=hGuQ8kuc9pgc9s8qqaq=dirpe0xb9q8qiLsFr0=vr0=vr0dc8meaabaqaciaacaGaaeqabaqabeGadaaakeaacqWGgbGrdaWgaaWcbaGaeGimaadabeaakiabcIcaOGqabiab=fgaHnaaBaaaleaacqWGPbqAaeqaaOGaeiykaKIaeyyyIO7aaabmaeaacqWG3bWDdaWgaaWcbaGaemyqae0aaSbaaWqaaiabdchaWbqabaaaleqaaaqaaiabdchaWjabg2da9iabigdaXaqaaiabd2gaTbqdcqGHris5aGGacOGae4hTdqMaeiikaGIaemyyae2aaSbaaSqaaiabdchaWjabcYcaSiabdMgaPbqabaGccqGGSaalcqGHsislcqGGPaqkaaa@4AA3@, where *δ*(*a*, *b*) = 1 if *a *= *b*, otherwise, *δ*(*a*, *b*) = 0, and '-' denotes a null. *F*_0_(**a**_*i*_) is the same as the null component of the frequency profile at **a**_*i*_, *i.e. F*_0_(**a**_*i*_) = fai,−
 MathType@MTEF@5@5@+=feaafiart1ev1aaatCvAUfKttLearuWrP9MDH5MBPbIqV92AaeXatLxBI9gBaebbnrfifHhDYfgasaacH8akY=wiFfYdH8Gipec8Eeeu0xXdbba9frFj0=OqFfea0dXdd9vqai=hGuQ8kuc9pgc9s8qqaq=dirpe0xb9q8qiLsFr0=vr0=vr0dc8meaabaqaciaacaGaaeqabaqabeGadaaakeaacqWGMbGzdaWgaaWcbaacbeGae8xyae2aaSbaaWqaaiabdMgaPbqabaWccqGGSaalcqGHsislaeqaaaaa@32DE@. We also define *F*_1_(*A*, *i*, *g*) as the sum of weights of sequences {*A*_*p*_} such that *a*_*p*,*i *_≠ '-' and the number of residues within segment *A*(*i *- *g *+ 1, *i*) aligned with the target gap is less than or equal to *x*_*c*_: F1(A,i,g)=∑p=1mwAp(1−δ(ap,i,−))Δ(ap,i,g)
 MathType@MTEF@5@5@+=feaafiart1ev1aaatCvAUfKttLearuWrP9MDH5MBPbIqV92AaeXatLxBI9gBaebbnrfifHhDYfgasaacH8akY=wiFfYdH8Gipec8Eeeu0xXdbba9frFj0=OqFfea0dXdd9vqai=hGuQ8kuc9pgc9s8qqaq=dirpe0xb9q8qiLsFr0=vr0=vr0dc8meaabaqaciaacaGaaeqabaqabeGadaaakeaacqWGgbGrdaWgaaWcbaGaeGymaedabeaakiabcIcaOiabdgeabjabcYcaSiabdMgaPjabcYcaSiabdEgaNjabcMcaPiabg2da9maaqadabaGaem4DaC3aaSbaaSqaaiabdgeabnaaBaaameaacqWGWbaCaeqaaaWcbeaaaeaacqWGWbaCcqGH9aqpcqaIXaqmaeaacqWGTbqBa0GaeyyeIuoakiabcIcaOiabigdaXiabgkHiTGGaciab=r7aKjabcIcaOiabdggaHnaaBaaaleaacqWGWbaCcqGGSaalcqWGPbqAaeqaaOGaeiilaWIaeyOeI0IaeiykaKIaeiykaKIaeuiLdqKaeiikaGIaemyyae2aaSbaaSqaaiabdchaWbqabaGccqGGSaalcqWGPbqAcqGGSaalcqWGNbWzcqGGPaqkaaa@5A81@, where Δ(*a*_*p*_, *i*, *g*) = 1 if the number of residues on the *p*-th row of *A*(*i *- *g *+ 1, *i*) is less than or equal to *x*_*c*_, otherwise, Δ(*a*_*p*_, *i*, *g*) = 0. Specifically, if ∑k=ii−g+1(1−δ(ap,k,−))≤xc
 MathType@MTEF@5@5@+=feaafiart1ev1aaatCvAUfKttLearuWrP9MDH5MBPbIqV92AaeXatLxBI9gBaebbnrfifHhDYfgasaacH8akY=wiFfYdH8Gipec8Eeeu0xXdbba9frFj0=OqFfea0dXdd9vqai=hGuQ8kuc9pgc9s8qqaq=dirpe0xb9q8qiLsFr0=vr0=vr0dc8meaabaqaciaacaGaaeqabaqabeGadaaakeaadaaeWaqaaiabcIcaOiabigdaXiabgkHiTGGaciab=r7aKjabcIcaOiabdggaHnaaBaaaleaacqWGWbaCcqGGSaalcqWGRbWAaeqaaOGaeiilaWIaeyOeI0IaeiykaKIaeiykaKIaeyizImQaemiEaG3aaSbaaSqaaiabdogaJbqabaaabaGaem4AaSMaeyypa0JaemyAaKgabaGaemyAaKMaeyOeI0Iaem4zaCMaey4kaSIaeGymaedaniabggHiLdaaaa@4A60@, then Δ(*a*_*p*_, *i*, *g*) = 1, otherwise, Δ(*a*_*p*_, *i*, *g*) = 0. Likewise, *F*_2_(*A*, *i*, *g*) is defined as the sum of weights of sequences {*A*_*p*_} such that *a*_*p*,*i *_≠ '-' and the number of residues within *A*(*i *- *g *+ 1, *i*) aligned with the target gap is greater than *x*_*c*_. Obviously,

*F*_0_(**a**_*i*_)+ *F*_1_(*A*, *i*, *g*) + *F*_2_(*A*, *i*, *g*) = 1.     (11)

Because there may exist gap states *g *and *g' *(0 ≤ *g *<*g' *≤ *i*) such that *F*_1_(*A*, *i*, *g*) = *F*_1_(*A*, *i*, *g'*), we need to store only distinct *F*_1_(*A*, *i*, *g*) values, the number of which is at most the number of rows of *A*, *m*. For actual calculations, we use an 'insertion length profile' associated with each column of a group. An insertion length profile of column **a**_*i*_, Fai
 MathType@MTEF@5@5@+=feaafiart1ev1aaatCvAUfKttLearuWrP9MDH5MBPbIqV92AaeXatLxBI9gBaebbnrfifHhDYfgasaacH8akY=wiFfYdH8Gipec8Eeeu0xXdbba9frFj0=OqFfea0dXdd9vqai=hGuQ8kuc9pgc9s8qqaq=dirpe0xb9q8qiLsFr0=vr0=vr0dc8meaabaqaciaacaGaaeqabaqabeGadaaakeaacqWGgbGrdaWgaaWcbaacbeGae8xyae2aaSbaaWqaaiabdMgaPbqabaaaleqaaaaa@30D1@ is expressed as Fai
 MathType@MTEF@5@5@+=feaafiart1ev1aaatCvAUfKttLearuWrP9MDH5MBPbIqV92AaeXatLxBI9gBaebbnrfifHhDYfgasaacH8akY=wiFfYdH8Gipec8Eeeu0xXdbba9frFj0=OqFfea0dXdd9vqai=hGuQ8kuc9pgc9s8qqaq=dirpe0xb9q8qiLsFr0=vr0=vr0dc8meaabaqaciaacaGaaeqabaqabeGadaaakeaacqWGgbGrdaWgaaWcbaacbeGae8xyae2aaSbaaWqaaiabdMgaPbqabaaaleqaaaaa@30D1@ = {(*i *- *g*_*k *_+ 1, *F*_1_(*A*, *i*, *g*_*k*_))}, where *g*_*k *_is a maximum gap state such that *F*_1_(*A*, *i*, *g*_*k*_) = *F*_1_(*A*, *i*, *g'*) for *g*_*k *_≤ *g' *<*g*_*k*+1_. Information about *F*_2_(*A*, *i*, *g*) does not need to be recorded, because it can be easily derived from *F*_0_(**a**_*i*_) and Fai
 MathType@MTEF@5@5@+=feaafiart1ev1aaatCvAUfKttLearuWrP9MDH5MBPbIqV92AaeXatLxBI9gBaebbnrfifHhDYfgasaacH8akY=wiFfYdH8Gipec8Eeeu0xXdbba9frFj0=OqFfea0dXdd9vqai=hGuQ8kuc9pgc9s8qqaq=dirpe0xb9q8qiLsFr0=vr0=vr0dc8meaabaqaciaacaGaaeqabaqabeGadaaakeaacqWGgbGrdaWgaaWcbaacbeGae8xyae2aaSbaaWqaaiabdMgaPbqabaaaleqaaaaa@30D1@ through equation 11. 

The second point raises the key problem: how to determine the 'tailored gap state' of the target gap, g^
 MathType@MTEF@5@5@+=feaafiart1ev1aaatCvAUfKttLearuWrP9MDH5MBPbIqV92AaeXatLxBI9gBaebbnrfifHhDYfgasaacH8akY=wiFfYdH8Gipec8Eeeu0xXdbba9frFj0=OqFfea0dXdd9vqai=hGuQ8kuc9pgc9s8qqaq=dirpe0xb9q8qiLsFr0=vr0=vr0dc8meaabaqaciaacaGaaeqabaqabeGadaaakeaacuWGNbWzgaqcaaaa@2E13@ = *g *- *s*, where *g *is the gap state of the target gap and *s *is the number of dynamic null columns to be removed for correct assignment of the relevant segment. For example, dynamic null columns aligned with **b**_5 _and **b**_11 _in Figure 1 are removed. To keep track of the dynamic gaps to be removed, we newly introduce the 'dynamic gap information' list. Each element of dynamic gap information is represented by (*p*, *l*), where *p *and *l *indicate the position and the length of a dynamic gap, respectively. For efficiency, the dynamic gap information list {(*p*_*k*_, *l*_*k*_)} is sorted in order of *p*_*k*_. The dynamic gap information of group *A *at (*i*, *j*), Di,j0
 MathType@MTEF@5@5@+=feaafiart1ev1aaatCvAUfKttLearuWrP9MDH5MBPbIqV92AaeXatLxBI9gBaebbnrfifHhDYfgasaacH8akY=wiFfYdH8Gipec8Eeeu0xXdbba9frFj0=OqFfea0dXdd9vqai=hGuQ8kuc9pgc9s8qqaq=dirpe0xb9q8qiLsFr0=vr0=vr0dc8meaabaqaciaacaGaaeqabaqabeGadaaakeaacqWGebardaqhaaWcbaGaemyAaKMaeiilaWIaemOAaOgabaGaeGimaadaaaaa@3270@(*A*), is recurrently calculated as follows. When **a**_*i *_and **b**_*j *_are aligned, Di,j0
 MathType@MTEF@5@5@+=feaafiart1ev1aaatCvAUfKttLearuWrP9MDH5MBPbIqV92AaeXatLxBI9gBaebbnrfifHhDYfgasaacH8akY=wiFfYdH8Gipec8Eeeu0xXdbba9frFj0=OqFfea0dXdd9vqai=hGuQ8kuc9pgc9s8qqaq=dirpe0xb9q8qiLsFr0=vr0=vr0dc8meaabaqaciaacaGaaeqabaqabeGadaaakeaacqWGebardaqhaaWcbaGaemyAaKMaeiilaWIaemOAaOgabaGaeGimaadaaaaa@3270@(*A*) is simply copied from Di−1,j−10
 MathType@MTEF@5@5@+=feaafiart1ev1aaatCvAUfKttLearuWrP9MDH5MBPbIqV92AaeXatLxBI9gBaebbnrfifHhDYfgasaacH8akY=wiFfYdH8Gipec8Eeeu0xXdbba9frFj0=OqFfea0dXdd9vqai=hGuQ8kuc9pgc9s8qqaq=dirpe0xb9q8qiLsFr0=vr0=vr0dc8meaabaqaciaacaGaaeqabaqabeGadaaakeaacqWGebardaqhaaWcbaGaemyAaKMaeyOeI0IaeGymaeJaeiilaWIaemOAaOMaeyOeI0IaeGymaedabaGaeGimaadaaaaa@362A@(*A*). Similarly, when **a**_*i *_is aligned with a null column, Di,j0
 MathType@MTEF@5@5@+=feaafiart1ev1aaatCvAUfKttLearuWrP9MDH5MBPbIqV92AaeXatLxBI9gBaebbnrfifHhDYfgasaacH8akY=wiFfYdH8Gipec8Eeeu0xXdbba9frFj0=OqFfea0dXdd9vqai=hGuQ8kuc9pgc9s8qqaq=dirpe0xb9q8qiLsFr0=vr0=vr0dc8meaabaqaciaacaGaaeqabaqabeGadaaakeaacqWGebardaqhaaWcbaGaemyAaKMaeiilaWIaemOAaOgabaGaeGimaadaaaaa@3270@(*A*) is copied from Di−1,j0
 MathType@MTEF@5@5@+=feaafiart1ev1aaatCvAUfKttLearuWrP9MDH5MBPbIqV92AaeXatLxBI9gBaebbnrfifHhDYfgasaacH8akY=wiFfYdH8Gipec8Eeeu0xXdbba9frFj0=OqFfea0dXdd9vqai=hGuQ8kuc9pgc9s8qqaq=dirpe0xb9q8qiLsFr0=vr0=vr0dc8meaabaqaciaacaGaaeqabaqabeGadaaakeaacqWGebardaqhaaWcbaGaemyAaKMaeyOeI0IaeGymaeJaeiilaWIaemOAaOgabaGaeGimaadaaaaa@344D@(*A*) or Di−1,j2
 MathType@MTEF@5@5@+=feaafiart1ev1aaatCvAUfKttLearuWrP9MDH5MBPbIqV92AaeXatLxBI9gBaebbnrfifHhDYfgasaacH8akY=wiFfYdH8Gipec8Eeeu0xXdbba9frFj0=OqFfea0dXdd9vqai=hGuQ8kuc9pgc9s8qqaq=dirpe0xb9q8qiLsFr0=vr0=vr0dc8meaabaqaciaacaGaaeqabaqabeGadaaakeaacqWGebardaqhaaWcbaGaemyAaKMaeyOeI0IaeGymaeJaeiilaWIaemOAaOgabaGaeGOmaidaaaaa@3451@(*A*) depending on the maximum operation of recurrent equation 3. When **b**_*j *_is aligned with a null column, Di,j0
 MathType@MTEF@5@5@+=feaafiart1ev1aaatCvAUfKttLearuWrP9MDH5MBPbIqV92AaeXatLxBI9gBaebbnrfifHhDYfgasaacH8akY=wiFfYdH8Gipec8Eeeu0xXdbba9frFj0=OqFfea0dXdd9vqai=hGuQ8kuc9pgc9s8qqaq=dirpe0xb9q8qiLsFr0=vr0=vr0dc8meaabaqaciaacaGaaeqabaqabeGadaaakeaacqWGebardaqhaaWcbaGaemyAaKMaeiilaWIaemOAaOgabaGaeGimaadaaaaa@3270@(*A*) is first copied from Di,j−10
 MathType@MTEF@5@5@+=feaafiart1ev1aaatCvAUfKttLearuWrP9MDH5MBPbIqV92AaeXatLxBI9gBaebbnrfifHhDYfgasaacH8akY=wiFfYdH8Gipec8Eeeu0xXdbba9frFj0=OqFfea0dXdd9vqai=hGuQ8kuc9pgc9s8qqaq=dirpe0xb9q8qiLsFr0=vr0=vr0dc8meaabaqaciaacaGaaeqabaqabeGadaaakeaacqWGebardaqhaaWcbaGaemyAaKMaeiilaWIaemOAaOMaeyOeI0IaeGymaedabaGaeGimaadaaaaa@344D@(*A*) or Di,j−13
 MathType@MTEF@5@5@+=feaafiart1ev1aaatCvAUfKttLearuWrP9MDH5MBPbIqV92AaeXatLxBI9gBaebbnrfifHhDYfgasaacH8akY=wiFfYdH8Gipec8Eeeu0xXdbba9frFj0=OqFfea0dXdd9vqai=hGuQ8kuc9pgc9s8qqaq=dirpe0xb9q8qiLsFr0=vr0=vr0dc8meaabaqaciaacaGaaeqabaqabeGadaaakeaacqWGebardaqhaaWcbaGaemyAaKMaeiilaWIaemOAaOMaeyOeI0IaeGymaedabaGaeG4mamdaaaaa@3453@(*A*), and then a new element is added to it or its last element is modified. Specifically, if the last element of Di,j0
 MathType@MTEF@5@5@+=feaafiart1ev1aaatCvAUfKttLearuWrP9MDH5MBPbIqV92AaeXatLxBI9gBaebbnrfifHhDYfgasaacH8akY=wiFfYdH8Gipec8Eeeu0xXdbba9frFj0=OqFfea0dXdd9vqai=hGuQ8kuc9pgc9s8qqaq=dirpe0xb9q8qiLsFr0=vr0=vr0dc8meaabaqaciaacaGaaeqabaqabeGadaaakeaacqWGebardaqhaaWcbaGaemyAaKMaeiilaWIaemOAaOgabaGaeGimaadaaaaa@3270@(*A*) is (*i*, *l*), it is modified to (*i*, *l *+ 1). Otherwise, a new element (*i*, 1) is added to Di,j0
 MathType@MTEF@5@5@+=feaafiart1ev1aaatCvAUfKttLearuWrP9MDH5MBPbIqV92AaeXatLxBI9gBaebbnrfifHhDYfgasaacH8akY=wiFfYdH8Gipec8Eeeu0xXdbba9frFj0=OqFfea0dXdd9vqai=hGuQ8kuc9pgc9s8qqaq=dirpe0xb9q8qiLsFr0=vr0=vr0dc8meaabaqaciaacaGaaeqabaqabeGadaaakeaacqWGebardaqhaaWcbaGaemyAaKMaeiilaWIaemOAaOgabaGaeGimaadaaaaa@3270@(*A*). The other dynamic gap information lists are obtained in a similar way.

It is worth mentioning that dynamic gap information Di,j0
 MathType@MTEF@5@5@+=feaafiart1ev1aaatCvAUfKttLearuWrP9MDH5MBPbIqV92AaeXatLxBI9gBaebbnrfifHhDYfgasaacH8akY=wiFfYdH8Gipec8Eeeu0xXdbba9frFj0=OqFfea0dXdd9vqai=hGuQ8kuc9pgc9s8qqaq=dirpe0xb9q8qiLsFr0=vr0=vr0dc8meaabaqaciaacaGaaeqabaqabeGadaaakeaacqWGebardaqhaaWcbaGaemyAaKMaeiilaWIaemOAaOgabaGaeGimaadaaaaa@3270@(*A*) and gap mediation profile Mi,j0
 MathType@MTEF@5@5@+=feaafiart1ev1aaatCvAUfKttLearuWrP9MDH5MBPbIqV92AaeXatLxBI9gBaebbnrfifHhDYfgasaacH8akY=wiFfYdH8Gipec8Eeeu0xXdbba9frFj0=OqFfea0dXdd9vqai=hGuQ8kuc9pgc9s8qqaq=dirpe0xb9q8qiLsFr0=vr0=vr0dc8meaabaqaciaacaGaaeqabaqabeGadaaakeaacqWGnbqtdaqhaaWcbaGaemyAaKMaeiilaWIaemOAaOgabaGaeGimaadaaaaa@3282@(*A*) contain information on dynamic gaps from different viewpoints. The information held in dynamic gap information is relevant to the group aligned with a gap in question (group *A *in the present example), while that maintained in a gap mediation profile is relevant to the group containing the gap (group *B *in the above example). In addition, each element of Di,j0
 MathType@MTEF@5@5@+=feaafiart1ev1aaatCvAUfKttLearuWrP9MDH5MBPbIqV92AaeXatLxBI9gBaebbnrfifHhDYfgasaacH8akY=wiFfYdH8Gipec8Eeeu0xXdbba9frFj0=OqFfea0dXdd9vqai=hGuQ8kuc9pgc9s8qqaq=dirpe0xb9q8qiLsFr0=vr0=vr0dc8meaabaqaciaacaGaaeqabaqabeGadaaakeaacqWGebardaqhaaWcbaGaemyAaKMaeiilaWIaemOAaOgabaGaeGimaadaaaaa@3270@(*A*) refers to a single dynamic gap, while that of Mi,j0
 MathType@MTEF@5@5@+=feaafiart1ev1aaatCvAUfKttLearuWrP9MDH5MBPbIqV92AaeXatLxBI9gBaebbnrfifHhDYfgasaacH8akY=wiFfYdH8Gipec8Eeeu0xXdbba9frFj0=OqFfea0dXdd9vqai=hGuQ8kuc9pgc9s8qqaq=dirpe0xb9q8qiLsFr0=vr0=vr0dc8meaabaqaciaacaGaaeqabaqabeGadaaakeaacqWGnbqtdaqhaaWcbaGaemyAaKMaeiilaWIaemOAaOgabaGaeGimaadaaaaa@3282@(*A*) records the total length of separate dynamic gaps inserted within or after a static gap. In the case of Figure 1, each element of D8,120
 MathType@MTEF@5@5@+=feaafiart1ev1aaatCvAUfKttLearuWrP9MDH5MBPbIqV92AaeXatLxBI9gBaebbnrfifHhDYfgasaacH8akY=wiFfYdH8Gipec8Eeeu0xXdbba9frFj0=OqFfea0dXdd9vqai=hGuQ8kuc9pgc9s8qqaq=dirpe0xb9q8qiLsFr0=vr0=vr0dc8meaabaqaciaacaGaaeqabaqabeGadaaakeaacqWGebardaqhaaWcbaGaeGioaGJaeiilaWIaeGymaeJaeGOmaidabaGaeGimaadaaaaa@3298@(*A*) = {(0, 1), (2, 2), (7, 1)} indicates the dynamic gaps of lengths 1, 2, and 1 inserted before column **a**_1_, after **a**_2_, and after **a**_7_, respectively, while that of M7,110
 MathType@MTEF@5@5@+=feaafiart1ev1aaatCvAUfKttLearuWrP9MDH5MBPbIqV92AaeXatLxBI9gBaebbnrfifHhDYfgasaacH8akY=wiFfYdH8Gipec8Eeeu0xXdbba9frFj0=OqFfea0dXdd9vqai=hGuQ8kuc9pgc9s8qqaq=dirpe0xb9q8qiLsFr0=vr0=vr0dc8meaabaqaciaacaGaaeqabaqabeGadaaakeaacqWGnbqtdaqhaaWcbaGaeG4naCJaeiilaWIaeGymaeJaeGymaedabaGaeGimaadaaaaa@32A6@(*A*) = {(0, 1), (2, 1), (7, 4) } means the total length of dynamic gaps inserted after the last non-null residue within or before the present column in *A*_1 _or *A*_4_, *A*_3_, and *A*_2_, respectively.

Given a dynamic gap information list *D*_*i*, *j*_(*A*) = {(*p*_*k*_, *l*_*k*_)} and a gap state *g*, we can easily derive the tailored gap state g^
 MathType@MTEF@5@5@+=feaafiart1ev1aaatCvAUfKttLearuWrP9MDH5MBPbIqV92AaeXatLxBI9gBaebbnrfifHhDYfgasaacH8akY=wiFfYdH8Gipec8Eeeu0xXdbba9frFj0=OqFfea0dXdd9vqai=hGuQ8kuc9pgc9s8qqaq=dirpe0xb9q8qiLsFr0=vr0=vr0dc8meaabaqaciaacaGaaeqabaqabeGadaaakeaacuWGNbWzgaqcaaaa@2E13@ with the following algorithm, in which *s *denotes the total number of dynamic gap columns to be removed and *t*_*k *_indicates the distance in the reverse direction from the current position *i *to the start position of the *k*-th dynamic gap inserted into *A*(1, *i*):

**Algorithm **getTailoredGapState(*D*_*i*,*j*_(*A*), *g*)

1. *s *← 0

2. for *k *← |*D*_*i*,*j*_(*A*)| down to 1

(a) *t*_*k *_← *s *+ *i *- *p*_*k*_+ *l*_*k*_

(b) if *t*_*k *_≤ *g*, then *s *← *s *+ *l*_*k*_

(c) else if *t*_*k *_- *l*_*k *_<*g*, then *s *← *s *+ *g *- (*t*_*k *_- *l*_*k*_)

(d) if *t*_*k *_≥ *g*, then return g^
 MathType@MTEF@5@5@+=feaafiart1ev1aaatCvAUfKttLearuWrP9MDH5MBPbIqV92AaeXatLxBI9gBaebbnrfifHhDYfgasaacH8akY=wiFfYdH8Gipec8Eeeu0xXdbba9frFj0=OqFfea0dXdd9vqai=hGuQ8kuc9pgc9s8qqaq=dirpe0xb9q8qiLsFr0=vr0=vr0dc8meaabaqaciaacaGaaeqabaqabeGadaaakeaacuWGNbWzgaqcaaaa@2E13@ ← *g *- *s*

3. retrun g^
 MathType@MTEF@5@5@+=feaafiart1ev1aaatCvAUfKttLearuWrP9MDH5MBPbIqV92AaeXatLxBI9gBaebbnrfifHhDYfgasaacH8akY=wiFfYdH8Gipec8Eeeu0xXdbba9frFj0=OqFfea0dXdd9vqai=hGuQ8kuc9pgc9s8qqaq=dirpe0xb9q8qiLsFr0=vr0=vr0dc8meaabaqaciaacaGaaeqabaqabeGadaaakeaacuWGNbWzgaqcaaaa@2E13@ ← *g *- *s*

The gap extension penalty for the target gap is then obtained by

*w*_*B*_{*u*_1_·*F*_1_(*A*, *i*, g^
 MathType@MTEF@5@5@+=feaafiart1ev1aaatCvAUfKttLearuWrP9MDH5MBPbIqV92AaeXatLxBI9gBaebbnrfifHhDYfgasaacH8akY=wiFfYdH8Gipec8Eeeu0xXdbba9frFj0=OqFfea0dXdd9vqai=hGuQ8kuc9pgc9s8qqaq=dirpe0xb9q8qiLsFr0=vr0=vr0dc8meaabaqaciaacaGaaeqabaqabeGadaaakeaacuWGNbWzgaqcaaaa@2E13@) + *u*_2_·*F*_2_(*A*, *i*, g^
 MathType@MTEF@5@5@+=feaafiart1ev1aaatCvAUfKttLearuWrP9MDH5MBPbIqV92AaeXatLxBI9gBaebbnrfifHhDYfgasaacH8akY=wiFfYdH8Gipec8Eeeu0xXdbba9frFj0=OqFfea0dXdd9vqai=hGuQ8kuc9pgc9s8qqaq=dirpe0xb9q8qiLsFr0=vr0=vr0dc8meaabaqaciaacaGaaeqabaqabeGadaaakeaacuWGNbWzgaqcaaaa@2E13@)},     (12)

where *w*_*B *_is the weight given to the sequence containing the target gap. Note that step 2c in this algorithm examines whether or not the dynamic gap in question is partially aligned with the target gap. For the example considered in Figure 1, *t*_3 _= 0 + 8 - 7 + 1 = 2, and *s *= *l*_3 _= 1 are calculated after the first iteration of steps 2 in this algorithm. In the second iteration, we obtain *t*_2 _= 1 + 8 - 2 + 2 = 9. Because *t*_2 _= 9 > *g *= 8 and *t*_2 _- *l*_2 _= 7 <*g *= 8, we can recognize that the dynamic gap is partially aligned with the target gap, and hence the number of dynamic null columns to be removed 8 - 7 = 1 is added to *s*. The gap extension penalty, *u*, summed over all the nulls on column **b**_*j *_can easily be calculated with the following algorithm:

1. *u *← 0

2. for *h *← 1 to |S^bj
 MathType@MTEF@5@5@+=feaafiart1ev1aaatCvAUfKttLearuWrP9MDH5MBPbIqV92AaeXatLxBI9gBaebbnrfifHhDYfgasaacH8akY=wiFfYdH8Gipec8Eeeu0xXdbba9frFj0=OqFfea0dXdd9vqai=hGuQ8kuc9pgc9s8qqaq=dirpe0xb9q8qiLsFr0=vr0=vr0dc8meaabaqaciaacaGaaeqabaqabeGadaaakeaacuWGtbWugaqcamaaBaaaleaaieqacqWFIbGydaWgaaadbaGaemOAaOgabeaaaSqabaaaaa@30FF@|

(a) g^
 MathType@MTEF@5@5@+=feaafiart1ev1aaatCvAUfKttLearuWrP9MDH5MBPbIqV92AaeXatLxBI9gBaebbnrfifHhDYfgasaacH8akY=wiFfYdH8Gipec8Eeeu0xXdbba9frFj0=OqFfea0dXdd9vqai=hGuQ8kuc9pgc9s8qqaq=dirpe0xb9q8qiLsFr0=vr0=vr0dc8meaabaqaciaacaGaaeqabaqabeGadaaakeaacuWGNbWzgaqcaaaa@2E13@ ← getTailoredGapState(*D*_*i*,*j*_(*A*), *g*_*h*_)

(b) *u *← *u *+ *f*_*h*_{*u*_1_·*F*_1_(*A*, *i*, gh_
 MathType@MTEF@5@5@+=feaafiart1ev1aaatCvAUfKttLearuWrP9MDH5MBPbIqV92AaeXatLxBI9gBaebbnrfifHhDYfgasaacH8akY=wiFfYdH8Gipec8Eeeu0xXdbba9frFj0=OqFfea0dXdd9vqai=hGuQ8kuc9pgc9s8qqaq=dirpe0xb9q8qiLsFr0=vr0=vr0dc8meaabaqaciaacaGaaeqabaqabeGadaaakeaadaqiaaqaaiabdEgaNnaaBaaaleaacqWGObaAaeqaaaGccaGLcmaaaaa@3054@) + *u*_2_·*F*_2_(*A*, *i*, gh_
 MathType@MTEF@5@5@+=feaafiart1ev1aaatCvAUfKttLearuWrP9MDH5MBPbIqV92AaeXatLxBI9gBaebbnrfifHhDYfgasaacH8akY=wiFfYdH8Gipec8Eeeu0xXdbba9frFj0=OqFfea0dXdd9vqai=hGuQ8kuc9pgc9s8qqaq=dirpe0xb9q8qiLsFr0=vr0=vr0dc8meaabaqaciaacaGaaeqabaqabeGadaaakeaadaqiaaqaaiabdEgaNnaaBaaaleaacqWGObaAaeqaaaGccaGLcmaaaaa@3054@)}

3. return *u*

where (*g*_*h*_, *f*_*h*_) is the *h*-th element of S^bj
 MathType@MTEF@5@5@+=feaafiart1ev1aaatCvAUfKttLearuWrP9MDH5MBPbIqV92AaeXatLxBI9gBaebbnrfifHhDYfgasaacH8akY=wiFfYdH8Gipec8Eeeu0xXdbba9frFj0=OqFfea0dXdd9vqai=hGuQ8kuc9pgc9s8qqaq=dirpe0xb9q8qiLsFr0=vr0=vr0dc8meaabaqaciaacaGaaeqabaqabeGadaaakeaacuWGtbWugaqcamaaBaaaleaaieqacqWFIbGydaWgaaadbaGaemOAaOgabeaaaSqabaaaaa@30FF@. Because we have already prepared running gap state profile S^bj
 MathType@MTEF@5@5@+=feaafiart1ev1aaatCvAUfKttLearuWrP9MDH5MBPbIqV92AaeXatLxBI9gBaebbnrfifHhDYfgasaacH8akY=wiFfYdH8Gipec8Eeeu0xXdbba9frFj0=OqFfea0dXdd9vqai=hGuQ8kuc9pgc9s8qqaq=dirpe0xb9q8qiLsFr0=vr0=vr0dc8meaabaqaciaacaGaaeqabaqabeGadaaakeaacuWGtbWugaqcamaaBaaaleaaieqacqWFIbGydaWgaaadbaGaemOAaOgabeaaaSqabaaaaa@30FF@, the computation is done in *O*(|*D*_*i*,*j*_(*A*)|·|S^bj
 MathType@MTEF@5@5@+=feaafiart1ev1aaatCvAUfKttLearuWrP9MDH5MBPbIqV92AaeXatLxBI9gBaebbnrfifHhDYfgasaacH8akY=wiFfYdH8Gipec8Eeeu0xXdbba9frFj0=OqFfea0dXdd9vqai=hGuQ8kuc9pgc9s8qqaq=dirpe0xb9q8qiLsFr0=vr0=vr0dc8meaabaqaciaacaGaaeqabaqabeGadaaakeaacuWGtbWugaqcamaaBaaaleaaieqacqWFIbGydaWgaaadbaGaemOAaOgabeaaaSqabaaaaa@30FF@|). To obtain the total gap extension penalty at each DP step, we must also consider the opposite situation where residues on **b**_*j *_are aligned with gaps on **a**_*i *_in a similar way.

### Doubly nested randomized iterative strategy

The doubly nested randomized iterative strategy involves refinement of alignment, phylogenetic tree, and pair weights until these are mutually consistent [9]. After preparation of an initial alignment with such progressive methods as the oligomer counting based method [8,10], this strategy refines the initial alignment as follows:

1. Calculate a distance matrix from the multiple alignment

2. Construct a phylogenetic tree from the distance matrix

3. Calculate pair weights from the phylogenetic tree

4. Iteratively refine the alignment using the phylogenetic tree and the pair weights

(a) Divide the alignment into two groups based on a randomly chosen branch of the tree

(b) Align these two groups using a group-to-group sequence alignment algorithm

(c) Repeat steps 4a to 4b until no better weighted SP score is obtained

5. Repeat steps 1 to 4 until the weighted SP score of the alignment does not improve anymore at step 4

## Results

### PRIME

We developed a program called PRIME (Profile-based Randomized Iteration Method). PRIME is written in ISO standard C++, implementing the doubly nested randomized iterative strategy similar to our previous MSA program, Prrn [9]. However, PRIME employs our proposed algorithm with a piecewise linear gap cost in contrast to Prrn that uses an affine gap cost. Another algorithmic difference between PRIME and Prrn is that the latter uses the candidate list paradigm in the group-to-group sequence alignment algorithm and the anchoring method, whereas the former adopts a simpler DP method without anchoring heuristics. The parameters of PRIME including selection of substitution matrix and gap cost parameters are optimized using an older BAliBASE, version 2.01 [22]. Because only about 20% of the sequences in BAliBASE version 3.0 [23] used for the test are common to those in BAliBASE version 2.01, we do not think that these parameters are over-fitted against BAliBASE version 3.0. Initial MSAs are constructed using a simple progressive method with the proposed group-to-group sequence alignment algorithm based on a distance matrix calculated from pairwise sequence alignment. The PRIME source code is provided as an additional file [see Additional file 1], and can be downloaded at PRIME website [24]. The future version of PRIME will be available at this site.

### Benchmarks

To evaluate the performance of PRIME and other MSA programs shown in Table 1, we execute two benchmarks: BAliBASE version 3.0 [22,23,25] and PREFAB version 4.0 [8]. In the case of PREFAB, we also test two global pairwise sequence alignment programs as controls: PSA_*piecewise *_and PSA_*affine*_. PSA_*piecewise *_and PSA_*affine *_use the piecewise linear gap cost and the affine gap cost, respectively.

**Table 1:** List of evaluated programs

program	version	option
PRIME_*piecewise*_		blosum62, *g*(*x*) = max{-(*x *+ 9), -(0.5*x *+ 21.5)}
PRIME_*affine*_		blosum62, *g*(*x*) = -(*x *+ 9)
Prrn [9]	3.4	-b2 -mblosum62 -u1 -v9
MAFFT* [15]	5.662	--maxiterate 1000 --localpair (L-INS-i)
ProbCons* [16]	1.09	default
T-Coffee* [27]	2.02	default
MUSCLE [8]	3.52	default
DIALIGN-T [28]	0.2.1	default
POA [5]	2	-do_global -do_progressive blosum80_trunc.mat
ClustalW [7]	1.83	default

Programs with * employ pairwise alignment information when calculating multiple alignment. Parameters or options of each program other than the gap penalty ones are chosen to obtain as accurate an alignment as possible.

#### BAliBASE

BAliBASE is categorized into five references according to the nature of sequences to be aligned (Table 2). Reference 1 is further divided into two sub-references based on sequence identities. Although the previous BAliBASE version 2.01 has been widely used, it had a problem that some sequences were trimmed off non-homologous regions [26]. Therefore, two test sets are prepared in BAliBASE version 3.0: full length set and homologous region set. Each sequence in the full length set is not trimmed off non-homologous regions, whereas the homologous region set consists of alignments of trimmed sequences and hence corresponds to the previous BAliBASE. However, reference 4 is excluded from the homologous region set due to its objective.

**Table 2:** BAliBASE version 3.0 contents

	no. of alignments	characteristic of alignment
Reference 1.1	37	phylogenetically equidistant (less than 20% identity)
Reference 1.2	42	phylogenetically equidistant (20 to 40% identity)
Reference 2	39	families including orphan sequences
Reference 3	29	equidistant families (less than 25% identity)
Reference 4	48	long N/C terminal extensions (excluded from homologous region set)
Reference 5	14	long internal insertions

#### Alignment evaluation based on BAliBASE

To evaluate alignment accuracy based on BAliBASE, we use sum-of-pairs and column scores [14]. The sum-of-pairs score (*SPS*) is defined as the proportion of correctly aligned residue pairs:

SPS=∑i=1ISPit∑j=1JSPjr,
 MathType@MTEF@5@5@+=feaafiart1ev1aaatCvAUfKttLearuWrP9MDH5MBPbIqV92AaeXatLxBI9gBaebbnrfifHhDYfgasaacH8akY=wiFfYdH8Gipec8Eeeu0xXdbba9frFj0=OqFfea0dXdd9vqai=hGuQ8kuc9pgc9s8qqaq=dirpe0xb9q8qiLsFr0=vr0=vr0dc8meaabaqaciaacaGaaeqabaqabeGadaaakeaacqWGtbWucqWGqbaucqWGtbWucqGH9aqpdaWcaaqaamaaqadabaGaem4uamLaemiuaa1aa0baaSqaaiabdMgaPbqaaiabdsha0baaaeaacqWGPbqAcqGH9aqpcqaIXaqmaeaacqWGjbqsa0GaeyyeIuoaaOqaamaaqadabaGaem4uamLaemiuaa1aa0baaSqaaiabdQgaQbqaaiabdkhaYbaaaeaacqWGQbGAcqGH9aqpcqaIXaqmaeaacqWGkbGsa0GaeyyeIuoaaaGccqGGSaalaaa@49A7@

where *I *and *J *are the number of columns of test and reference alignments, respectively. SPit
 MathType@MTEF@5@5@+=feaafiart1ev1aaatCvAUfKttLearuWrP9MDH5MBPbIqV92AaeXatLxBI9gBaebbnrfifHhDYfgasaacH8akY=wiFfYdH8Gipec8Eeeu0xXdbba9frFj0=OqFfea0dXdd9vqai=hGuQ8kuc9pgc9s8qqaq=dirpe0xb9q8qiLsFr0=vr0=vr0dc8meaabaqaciaacaGaaeqabaqabeGadaaakeaacqWGtbWucqWGqbaudaqhaaWcbaGaemyAaKgabaGaemiDaqhaaaaa@31FD@ is defined as:

SPit=∑1≤m<n≤Npi(m,n).
 MathType@MTEF@5@5@+=feaafiart1ev1aaatCvAUfKttLearuWrP9MDH5MBPbIqV92AaeXatLxBI9gBaebbnrfifHhDYfgasaacH8akY=wiFfYdH8Gipec8Eeeu0xXdbba9frFj0=OqFfea0dXdd9vqai=hGuQ8kuc9pgc9s8qqaq=dirpe0xb9q8qiLsFr0=vr0=vr0dc8meaabaqaciaacaGaaeqabaqabeGadaaakeaacqWGtbWucqWGqbaudaqhaaWcbaGaemyAaKgabaGaemiDaqhaaOGaeyypa0ZaaabuaeaacqWGWbaCdaWgaaWcbaGaemyAaKgabeaaaeaacqaIXaqmcqGHKjYOcqWGTbqBcqGH8aapcqWGUbGBcqGHKjYOcqWGobGtaeqaniabggHiLdGccqGGOaakcqWGTbqBcqGGSaalcqWGUbGBcqGGPaqkcqGGUaGlaaa@47A8@

If aligned residue pair *a*_*mi *_and *a*_*ni *_of the test alignment also exists in the reference alignment, *P*_*i*_(*m*, *n*) = 1. Otherwise, *p*_*i*_(*m*, *n*) = 0. SPjr
 MathType@MTEF@5@5@+=feaafiart1ev1aaatCvAUfKttLearuWrP9MDH5MBPbIqV92AaeXatLxBI9gBaebbnrfifHhDYfgasaacH8akY=wiFfYdH8Gipec8Eeeu0xXdbba9frFj0=OqFfea0dXdd9vqai=hGuQ8kuc9pgc9s8qqaq=dirpe0xb9q8qiLsFr0=vr0=vr0dc8meaabaqaciaacaGaaeqabaqabeGadaaakeaacqWGtbWucqWGqbaudaqhaaWcbaGaemOAaOgabaGaemOCaihaaaaa@31FB@ is the total number of aligned pairs on column *j *of the reference alignment. The column score (*CS*) represents the proportion of correctly aligned columns:

CS=1J∑i=1Ici.
 MathType@MTEF@5@5@+=feaafiart1ev1aaatCvAUfKttLearuWrP9MDH5MBPbIqV92AaeXatLxBI9gBaebbnrfifHhDYfgasaacH8akY=wiFfYdH8Gipec8Eeeu0xXdbba9frFj0=OqFfea0dXdd9vqai=hGuQ8kuc9pgc9s8qqaq=dirpe0xb9q8qiLsFr0=vr0=vr0dc8meaabaqaciaacaGaaeqabaqabeGadaaakeaacqWGdbWqcqWGtbWucqGH9aqpdaWcaaqaaiabigdaXaqaaiabdQeakbaadaaeWbqaaiabdogaJnaaBaaaleaacqWGPbqAaeqaaaqaaiabdMgaPjabg2da9iabigdaXaqaaiabdMeajbqdcqGHris5aOGaeiOla4caaa@3C74@

If the column of the test alignment is identical to the *i*-th column of the reference alignment, *c*_*i *_= 1.

Otherwise, *c*_*i *_= 0.

#### PREFAB

PREFAB is another MSA benchmark. Each alignment of PREFAB is generated automatically, while that of BAliBASE is constructed by human expertise. PREFAB consists of three data sets: main, long gap, and weighting sets. The main set corresponds to the previous PREFAB version 3.0, which is not categorized unlike BAliBASE. Each alignment of the long gap set, a subset of the main set, contains one or more gaps whose lengths are more than 10. The weighting set involves alignments each of which includes more sequences of one sub-family than that of the other sub-families. Whereas each reference alignment of BAliBASE is provided as an MSA, each reference alignment of PREFAB is provided as a pairwise alignment of a pair of PDB sequences of known structures.

#### Alignment evaluation based on PREFAB

For alignment evaluation of PREFAB, the quality score is employed, which measures only two PDB sequences within each alignment. The quality score (*QS*) is the ratio of correctly aligned residue pairs of the reference pairwise alignment: QS=1J∑i=1Jpi
 MathType@MTEF@5@5@+=feaafiart1ev1aaatCvAUfKttLearuWrP9MDH5MBPbIqV92AaeXatLxBI9gBaebbnrfifHhDYfgasaacH8akY=wiFfYdH8Gipec8Eeeu0xXdbba9frFj0=OqFfea0dXdd9vqai=hGuQ8kuc9pgc9s8qqaq=dirpe0xb9q8qiLsFr0=vr0=vr0dc8meaabaqaciaacaGaaeqabaqabeGadaaakeaacqWGrbqucqWGtbWucqGH9aqpdaWcaaqaaiabigdaXaqaaiabdQeakbaadaaeWaqaaiabdchaWnaaBaaaleaacqWGPbqAaeqaaaqaaiabdMgaPjabg2da9iabigdaXaqaaiabdQeakbqdcqGHris5aaaa@3B7E@, where *J *is the number of residue pairs in the reference alignment. If the residue pair of the test alignment is also aligned in the reference alignment, *p*_*i *_= 1. Otherwise, *p*_*i *_= 0. Note that if the reference alignment is pairwise alignment, quality score, sum-of-pairs score, and column score have the same value.

### Results of BAliBASE benchmark test

#### Full length set

**Figure 2:**
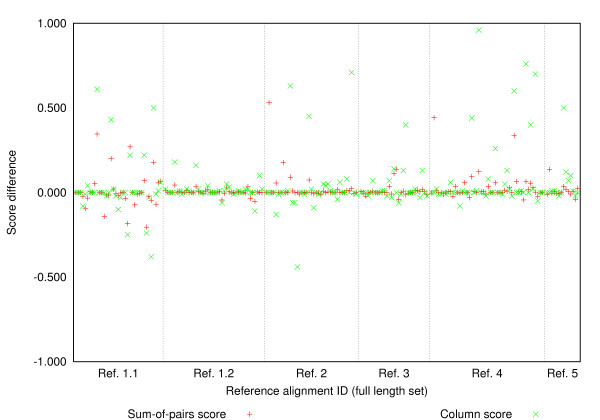
**Score differences between PRIME**_***piecewise ***_**and PRIME**_***affine ***_**on full length set**. The horizontal axis denotes reference alignment ID, and the vertical axis, the difference in sum-of-pairs or column scores on respective alignments of the full length set using PRIME_*piecewise *_and PRIME_*affine*_. A positive difference score of an alignment is an indication that PRIME_*piecewise *_shows better performance than PRIME_*affine *_for the alignment, and vice versa.

The average sum-of-pairs and column scores of the full length set are shown in Tables 3 and 4, respectively. The last columns of both tables are the rank sum of the Friedman test. The program with the smallest rank sum means that the program consistently constructs the most accurate alignments even if it does not achieve the largest average score. The *p*-values of the Friedman test are shown in the additional file [see Additional file 2]. The Friedman test indicates that the tested programs are classified into three groups according to their performances. The most accurate group consists of PRIME_*piecewise*_, PRIME_*affine*_, MAFFT, ProbCons, and T-Coffee. The second most accurate one is Prrn and MUSCLE. The accuracies of DIALIGN-T, POA, and ClustalW are comparable to each other but are significantly lower than those of Prrn and MUSCLE. Figure 2 shows the performance difference between PRIME_*piecewise *_and PRIME_*affine*_. In this figure, we plot the difference in alignment scores of PRIME_*piecewise *_and PRIME_*affine*_. A positive difference score means that PRIME_*piecewise *_constructs more accurate alignments than PRIME_*affine*_, and *vice versa*. Although the difference is not statistically significant, PRIME_*piecewise *_shows better performance than PRIME_*affine*_, as expected.

**Table 3:** Average sum-of-pairs scores of full length set

	Ref. 1.1	Ref. 1.2	Ref. 2	Ref. 3	Ref. 4	Ref. 5	Overall	Ranksum
PRIME_*piecewise*_	0.643	0.933	0.922	0.859	0.910	0.882	0.861	809
PRIME_*affine*_	0.635	0.931	0.898	0.851	0.882	0.871	0.846	912
Prrn	0.574	0.923	0.901	0.820	0.859	0.821	0.821	1055
MAFFT	0.671	0.938	0.923	0.852	0.918	0.892	0.868	656
ProbCons	0.648	0.942	0.905	0.835	0.887	0.879	0.851	764
T-Coffee	0.613	0.933	0.916	0.826	0.900	0.858	0.846	884
MUSCLE	0.570	0.909	0.888	0.808	0.857	0.839	0.815	1260
DIALIGN-T	0.489	0.888	0.859	0.744	0.817	0.780	0.768	1668
POA	0.474	0.857	0.857	0.733	0.805	0.754	0.753	1804
ClustalW	0.497	0.864	0.848	0.722	0.786	0.713	0.748	1682

Each column shows average sum-of-pairs scores using all alignments of each reference of the full length set. Overall and Ranksum columns show the average sum-of-pairs scores and the rank sum of the Friedman test using all alignment of the whole full length set, respectively. A smaller rank sum means better accuracy.

**Table 4:** Average column scores of full length set

	Ref. 1.1	Ref. 1.2	Ref. 2	Ref. 3	Ref. 4	Ref. 5	Overall	Ranksum
PEIME_*piecewise*_	0.416	0.839	0.445	0.566	0.573	0.552	0.572	846
PRIME_*affine*_	0.391	0.826	0.413	0.539	0.483	0.496	0.531	958
Prrn	0.334	0.791	0.406	0.469	0.491	0.411	0.499	1080
MAFFT	0.449	0.839	0.436	0.560	0.607	0.544	0.583	759
ProbCons	0.401	0.851	0.374	0.462	0.530	0.509	0.532	847
T-Coffee	0.324	0.832	0.384	0.459	0.563	0.534	0.525	1017
MUSCLE	0.313	0.795	0.343	0.380	0.460	0.408	0.465	1246
DIALIGN-T	0.246	0.723	0.290	0.347	0.462	0.389	0.423	1554
POA	0.224	0.678	0.265	0.343	0.413	0.323	0.389	1690
ClustalW	0.221	0.707	0.219	0.271	0.404	0.237	0.368	1497

Each column shows average column scores using all alignments of each reference of the full length set. Overall and Ranksum columns show the average column scores and the rank sum of the Friedman test using all alignment of the whole full length set, respectively. A smaller rank sum means better accuracy.

#### Homologous region set

**Figure 3:**
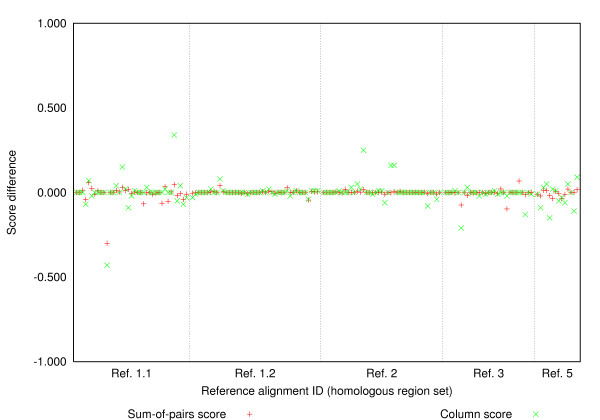
**Score differences between PRIME**_***piecewise ***_**and PRIME**_***affine ***_**on homologous region set**. The horizontal axis denotes reference alignment ID, and the vertical axis, the difference in sum-of-pairs or column scores on respective alignments of the homologous region set using PRIME_*piecewise *_and PRIME_*affine*_. A positive difference score of an alignment is an indication that PRIME_*piecewise *_shows better performance than PRIME_*affine *_for the alignment, and vice versa.

The average sum-of-pairs and column scores of the homologous region set are shown in Tables 5 and 6, respectively. The *p*-values of the Friedman test are shown in the additional file [see Additional file 3]. Unlike the results of the full length set, little difference in accuracy was detected between PRIME_*piecewise *_and PRIME_*affine*_. Figure 3 shows the performance difference between them. Because the terminal non-homologous regions trimmed off in the homologous region set are usually long, for the full length set, the piecewise linear gap cost treats the long terminal gaps more effectively than the affine gap cost, and hence PRIME_*piecewise *_shows better performance than PRIME_*affine*_. However, because the difference between these gap costs is relatively small in the homologous region set, PRIME_*piecewise *_and PRIME_*affine *_show similar performance. The relative performance of the nine programs examined is nearly the same as that of the full length set in a statistical sense.

**Table 5:** Average sum-of-pairs scores of homologous region set

	Ref. 1.1	Ref. 1.2	Ref. 2	Ref. 3	Ref. 5	Overall	Ranksum
PRIME_*piecewise*_	0.772	0.940	0.955	0.903	0.891	0.894	613
PRIME_*affine*_	0.781	0.938	0.954	0.907	0.896	0.897	634
Prrn	0.763	0.936	0.954	0.894	0.887	0.889	698
MAFFT	0.753	0.940	0.946	0.890	0.897	0.886	654
ProbCons	0.788	0.953	0.953	0.910	0.907	0.904	489
T-Coffee	0.704	0.939	0.940	0.878	0.888	0.870	821
MUSCLE	0.735	0.931	0.943	0.882	0.870	0.875	907
DIALIGN-T	0.573	0.901	0.897	0.793	0.821	0.798	1406
POA	0.634	0.877	0.923	0.822	0.800	0.816	1370
ClustalW	0.664	0.905	0.922	0.816	0.788	0.827	1263

Each column shows average sum-of-pairs scores using all alignments of each reference of the homologous region set. Overall and Ranksum columns show the average sum-of-pairs scores and the rank sum of the Friedman test using all alignment of the whole homologous region set, respectively. A smaller rank sum means better accuracy.

**Table 6:** Average column scores of homologous region set

	Ref. 1.1	Ref. 1.2	Ref. 2	Ref. 3	Ref. 5	Overall	Ranksum
PEIME_*piecewise*_	0.588	0.849	0.595	0.636	0.575	0.665	640
PRIME_*affine*_	0.589	0.847	0.582	0.648	0.593	0.666	640
Prrn	0.561	0.834	0.601	0.630	0.558	0.654	708
MAFFT	0.552	0.846	0.532	0.631	0.578	0.640	670
ProbCons	0.591	0.875	0.540	0.625	0.583	0.658	548
T-Coffee	0.476	0.840	0.491	0.625	0.540	0.607	822
MUSCLE	0.496	0.823	0.496	0.574	0.501	0.596	946
DIALIGN-T	0.338	0.761	0.370	0.452	0.429	0.485	1339
POA	0.390	0.712	0.424	0.459	0.371	0.493	1348
ClustalW	0.416	0.791	0.443	0.475	0.394	0.529	1193

Each column shows average column scores using all alignments of each reference of the homologous region set. Overall and Ranksum columns show the average column scores and the rank sum of the Friedman test using all alignment of the whole homologous region set, respectively. A smaller rank sum means better accuracy.

#### Effects of non-homologous regions

**Figure 4:**
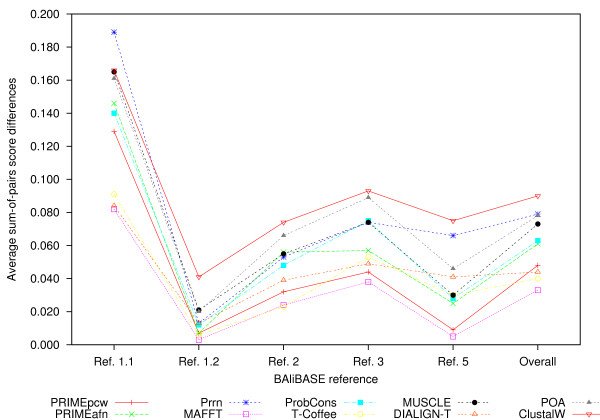
**Average sum-of-pairs score differences between full length and homologous region sets**. Each point means average sum-of-pairs score difference in respective alignments on each reference of the full length and homologous region sets. PRIME_*pcw *_denotes PRIME_*piecewise*_, and PRIME_*afn*_, PRIME_*affine*_. The smaller absolute value of a score indicates that the introduction of long terminal indels less affects the alignment accuracy of a program.

**Figure 5:**
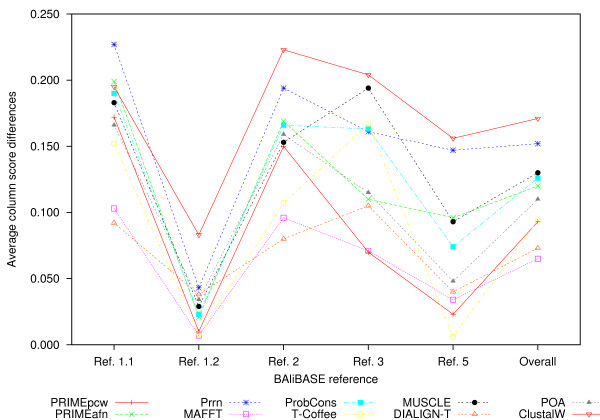
**Average column score differences between full length and homologous region sets**. Each point means average column score difference in respective alignments on each reference of the full length and homologous region sets. PRIME_*pcw *_denotes PRIME_*piecewise*_, and PRIME_*afn*_, PRIME_*affine*_. The smaller absolute value of a score indicates that the introduction of long terminal indels less affects the alignment accuracy of a program.

We examine the effects of non-homologous regions in more detail. The critical difference between the full length and homologous region sets is the existence of non-homologous regions at N/C terminals. Therefore, the difference in alignment scores obtained by the same program for the corresponding members in the full length and homologous region sets indicates to what extent the program properly deals with terminal gaps. Figures 4 and 5 show the average difference of sum-of-pairs and column scores between the full length and homologous region sets. Each difference score is calculated by subtracting the alignment score of the full length set from that of the homologous region set. A positive difference score means that the non-homologous regions adversely affect alignment accuracy, whereas a negative score indicates improvement in alignment accuracy due to the presence of such regions. If the score difference is close to 0, the program is considered to be robust against the non-homologous regions. The results indicate that PRIME_*piecewise *_is less affected by such regions than PRIME_*affine*_. This follows the general tendency that terminal gaps reduce more significantly the accuracy of global alignment programs including Prrn, MUSCLE, POA, and ClustalW than that of MAFFT, ProbCons, and T-Coffee that incorporate local alignment information in some ways. These observations indicate that PRlME_*piecewise *_deals with terminal gaps better than conventional global MSA programs, although not as well as those incorporating local alignment information.

### Results of PREFAB benchmark test

The average quality scores of the three sets of PREFAB are shown in Table 7. The overall tendencies of relative performances and the Friedman tests of the main and long gap sets are nearly the same as those of BAliBASE. However, in the case of the weighting set, all programs except POA are comparable to each other. Because POA does not use sequence weights, 'biased sub-family composition' might adversely affect the performance of POA compared with the other programs.

**Table 7:** Average quality scores of PREFAB

	Main		Weighting		Long gap	
	QS	Ranksum	QS	Ranksum	QS	Ranksum

PRIME_*piecewise*_	0.721	8151	0.649	588	0.658	1408
PRIME_*affine*_	0.718	8355	0.637	617	0.651	1504
Prrn	0.722	8120	0.624	621	0.653	1455
MAFFT	0.722	7744	0.639	585	0.660	1352
ProbCons	0.705	8659	0.620	594	0.637	1443
T-Coffee	0.700	9126	0.627	584	0.631	1640
MUSCLE	0.680	10446	0.607	642	0.596	1918
DIALIGN-T	0.621	13277	0.587	754	0.541	2506
POA	0.603	14662	0.554	868	0.513	2789
ClustalW	0.617	12952	0.603	650	0.519	2583
PSA_*piecewise*_	0.591	14525	0.638	627	0.498	2804
PSA_*affine*_	0.581	14789	0.621	670	0.489	2856

Each QS and Ranksum columns show the average quality scores and the rank sum of the Friedman test using quality scores on all alignments of each reference set, respectively. A smaller rank sum means better accuracy.

### Computation time

The computation time of each program for executing the benchmarks is compiled in Table 8. The computer we used is Pentium3 933 MHz with 1 GB memory, running on RedHat Linux 7.3. PRIME_*piecewise *_and PRIME_*affine *_are somewhat slower than most programs tested. The computational speed would be significantly improved by incorporating anchoring heuristics and refining source codes.

**Table 8:** Computation time

	BAliBASE	PREFAB
PRIME_*piecewise*_	9.4 × 10^5^	5.5 × 10^5^
PRIME_*affine*_	4.9 × 10^5^	4.3 × 10^5^
Prrn	6.8 × 10^5^	1.9 × 10^5^
MAFFT	1.9 × 10^4^	2.7 × 10^4^
ProbCons	6.4 × 10^4^	1.9 × 10^5^
T-Coffee	7.2 × 10^5^	2.0 × 10^6^
MUSCLE	7.9 × 10^3^	1.6 × 10^4^
DIALIGN-T	3.0 × 10^4^	1.2 × 10^5^
POA	1.0 × 10^4^	2.6 × 10^4^
ClustalW	8.3 × 10^3^	2.7 × 10^4^

BAliBASE column shows total times (sec.) of constructing all alignments of the full length and homologous region sets by each program, while PREFAB column, those of calculating whole alignments of the main and weighting sets only.

## Discussions and Conclusion

The group-to-group sequence alignment algorithm is the key to most heuristic MSA algorithms. Although many group-to-group sequence alignment algorithms focus on position-specific gap opening penalties [7,8,10,12], they use essentially a constant gap extension penalty similar to that of the affine gap cost. For global MSA algorithms, use of the constant gap extension penalty could lead to deterioration of alignment accuracy when some of the sequences to be aligned have long indels. To our knowledge, POA version 2 [5] is the sole precedent that incorporates length-dependent gap extension penalties into the group-to-group sequence alignment algorithm. Examination of POA with various options indicated that length-dependent gap extension penalties with global alignment strategy are effective to improve alignment accuracy when some of the sequences to be aligned have long indels (data not shown).

In this paper, we proposed a novel group-to-group sequence alignment algorithm with the piecewise linear gap cost, and developed a program called PRIME. The advantage of using the piecewise linear gap cost is that this gap cost more accurately models the occurrence of long gap in a simple way than other gap cost does. As a result of BAliBASE benchmark test, PRIME achieved alignment accuracies comparable to the most accurate programs available today including L-INS-i of MAFFT, ProbCons, and T-Coffee. Unlike others, PRIME does not rely on pairwise alignment information. This implies that the introduction of length-dependent gap extension penalties could contribute to improving the alignment accuracy even when pairwise alignment information is not used.

It should be noted that our proposed algorithm has two inherent drawbacks. First, it is considerably slower than many popular algorithms. Second, selecting the parameters of the piecewise linear gap cost is somewhat more complicated than that of the affine gap cost. However, these drawbacks would not be serious enough to reduce the advantages of our proposed algorithm and PRIME. Unlike most popular algorithms, PRIME can circumvent the time-consuming process for obtaining pairwise alignment information, and hence it is theoretically advantageous for aligning a large number of sequences. Our preliminary examination confirmed the expected dependency of computational time on the number of sequences to be aligned. However, the current version of PRIME is still slower than most of other programs over the examined range of the number of sequences (data not shown). One of the reasons is that the current PRIME does not use any heuristics, such as anchoring or grouping method used in Prrn, for reducing the computation. To improve calculation speed of PRIME without a loss of accuracy, we are attempting to incorporate these heuristics. To further improve alignment accuracy, we will investigate several problems including the conditions under which PRIME_*affine *_constructs more accurate alignment than PRIME_*piecewise*_, the potential of other objective functions, and the effect of incorporating pairwise alignment information.

## Availability and Requirements

Project name: PRIME project

Project home page: 

Operating system(s): Platform independent

Programming language: C++

Licence: GNU GPL version 2 or later

Any restrictions to use by non-academics: None

## Authors' contributions

SY devised the proposed algorithms, implemented PRIME, carried out the benchmark test and its evaluation, and drafted the manuscript. OG conceived of the study, devised the proposed algorithms, helped to implement PRIME, and drafted the manuscript. HY participated in the design and coordination of the study, and helped to implement PRIME. All authors read and approved the final manuscript.

## Supplementary Material

Additional File 1 **PRIME source code**. This tar.gz archive includes the source files of PRIME. To compile PRIME, one can check 'INSTALL' and 'Makefile' in the archive. Although 'Makefile' basically assumes GNU make and g+ +, another compiler can be used with a few modification of 'Makefile'. Click here for file

Additional File 2 ***p*-values of the Friedman test of full length set**. Each value is *p*-value of the Friedman test, indicating the significance of a difference in performance between programs of a row and a column. The upper right and lower left *p*-values are calculated using sum-of-pairs and column scores on all alignments of the whole full length set, respectively. PRIME_*pcw *_denotes PRIME_*piecewise*_, and PRIME_*afn*_, PRIME_*affine*_. The respective signs + and - denote that a program of a row performs significantly better and worse than that of a column. Click here for file

Additional File 3 ***p*-values of the Friedman test of homologous region set**. Each value is *p*-value of the Friedman test, indicating the significance of a difference in performance between programs of a row and a column. The upper right and lower left *p*-values are calculated using sum-of-pairs and column scores on all alignments of the whole homologous region set, respectively. PRIME_*pcw *_denotes PRIME_*piecewise*_, and PRIME_*afn*_, PRIME_*affine*_. The respective signs + and - denote that a program of a row performs significantly better and worse than that of a column. Click here for file
