# Supplementary material for: Improvement in accuracy of multiple sequence alignment using novel group-to-group sequence alignment algorithm with piecewise linear gap cost
Source: BMC Bioinformatics. 2006 Dec 1;7:524. doi: 10.1186/1471-2105-7-524 (PMC1769516; doi:10.1186/1471-2105-7-524)
Supplement: Additional File 2 — p-values of the Friedman test of full length set. Each value is p-value of the Friedman test, indicating the significance of a difference in performance between programs of a row and a column. The upper right and lower left p-values are calculated using sum-of-pairs and column scores on all alignments of the whole full length set, respectively. PRIMEpcw denotes PRIMEpiecewise, and PRIMEafn, PRIMEaffine. The respective signs + and - denote that a program of a row performs significantly better and worse than that of a column. [file 1471-2105-7-524-S2.pdf]

	PRIME$_{pcw}$	PRIME$_{afn}$	Prrn	MAFFT	ProbCons	T-Coffee	MUSCLE	DIALIGN-T	POA	ClustalW
PRIME$_{pcw}$		0.97	0.068	0.73	1.0	1.0	$+2.2 \times 10^{-8}$	$+ < 10^{-10}$	$+ < 10^{-10}$	$+ < 10^{-10}$
PRIME$_{afn}$	0.92		0.80	-4.5×10^{-2}	0.76	1.0	$+2.1 \times 10^{-4}$	$+ < 10^{-10}$	$+ < 10^{-10}$	$+ < 10^{-10}$
Prrn	0.050	0.87		-3.6×10^{-6}	-7.8×10^{-3}	0.56	0.27	$+ < 10^{-10}$	$+ < 10^{-10}$	$+ < 10^{-10}$
MAFFT	0.98	0.19	$+2.0 \times 10^{-4}$		0.96	0.14	$+ < 10^{-10}$	$+ < 10^{-10}$	$+ < 10^{-10}$	$+ < 10^{-10}$
ProbCons	1.0	0.92	0.051	0.98		0.93	$+1.4 \times 10^{-10}$	$+ < 10^{-10}$	$+ < 10^{-10}$	$+ < 10^{-10}$
T-Coffee	0.44	1.0	1.0	-1.4×10^{-2}	0.44		$+2.2 \times 10^{-5}$	$+ < 10^{-10}$	$+ < 10^{-10}$	$+ < 10^{-10}$
MUSCLE	-1.4×10^{-7}	-2.4×10^{-3}	0.48	$- < 10^{-10}$	-1.4×10^{-7}	0.063		$+1.6 \times 10^{-6}$	$+ < 10^{-10}$	$+4.4 \times 10^{-7}$
DIALIGN-T	$- < 10^{-10}$	$- < 10^{-10}$	$- < 10^{-10}$	$- < 10^{-10}$	$- < 10^{-10}$	$- < 10^{-10}$	-5.2×10^{-4}		0.84	1.0
POA	$- < 10^{-10}$	$- < 10^{-10}$	$- < 10^{-10}$	$- < 10^{-10}$	$- < 10^{-10}$	$- < 10^{-10}$	-6.9×10^{-10}	0.76		0.92
ClustalW	$- < 10^{-10}$	$- < 10^{-10}$	-1.9×10^{-8}	$- < 10^{-10}$	$- < 10^{-10}$	$- < 10^{-10}$	-2.0×10^{-2}	1.0	0.24	
