# Supplementary material for: Improvement in accuracy of multiple sequence alignment using novel group-to-group sequence alignment algorithm with piecewise linear gap cost
Source: BMC Bioinformatics. 2006 Dec 1;7:524. doi: 10.1186/1471-2105-7-524 (PMC1769516; doi:10.1186/1471-2105-7-524)
Supplement: Additional File 3 — p-values of the Friedman test of homologous region set. Each value is p-value of the Friedman test, indicating the significance of a difference in performance between programs of a row and a column. The upper right and lower left p-values are calculated using sum-of-pairs and column scores on all alignments of the whole homologous region set, respectively. PRIMEpcw denotes PRIMEpiecewise, and PRIMEafn, PRIMEaffine. The respective signs + and - denote that a program of a row performs significantly better and worse than that of a column. [file 1471-2105-7-524-S3.pdf]

	PRIME_{pcw}	PRIME_{afn}	Prrn	MAFFT	ProbCons	T-Coffee	MUSCLE	DIALIGN-T	POA	ClustalW
PRIME_{pcw}		1.0	0.98	1.0	0.81	0.092	$+4.6 \times 10^{-4}$	$+ < 10^{-10}$	$+ < 10^{-10}$	$+ < 10^{-10}$
PRIME_{afn}	1.0		1.0	1.0	0.61	0.21	$+2.2 \times 10^{-3}$	$+ < 10^{-10}$	$+ < 10^{-10}$	$+ < 10^{-10}$
Prrn	1.0	1.0		1.0	0.088	0.81	0.088	$+ < 10^{-10}$	$+ < 10^{-10}$	$+ < 10^{-10}$
MAFFT	1.0	1.0	1.0		0.40	0.38	$+8.5 \times 10^{-3}$	$+ < 10^{-10}$	$+ < 10^{-10}$	$+ < 10^{-10}$
ProbCons	0.96	0.97	0.44	0.81		$+1.7 \times 10^{-5}$	$+1.1 \times 10^{-9}$	$+ < 10^{-10}$	$+ < 10^{-10}$	$+ < 10^{-10}$
T-Coffee	0.23	0.22	0.86	0.51	-1.6×10^{-3}		0.98	$+ < 10^{-10}$	$+ < 10^{-10}$	$+ < 10^{-10}$
MUSCLE	-1.2×10^{-4}	-1.1×10^{-4}	-1.6×10^{-2}	-1.3×10^{-3}	-6.6×10^{-9}	0.79		$+ < 10^{-10}$	$+ < 10^{-10}$	$+1.5 \times 10^{-6}$
DIALIGN-T	$- < 10^{-10}$	$- < 10^{-10}$	$- < 10^{-10}$	$- < 10^{-10}$	$- < 10^{-10}$	$- < 10^{-10}$	-1.3×10^{-8}		1.0	0.63
POA	$- < 10^{-10}$	$- < 10^{-10}$	$- < 10^{-10}$	$- < 10^{-10}$	$- < 10^{-10}$	$- < 10^{-10}$	-4.5×10^{-9}	1.0		0.92
ClustalW	$- < 10^{-10}$	$- < 10^{-10}$	$- < 10^{-10}$	$- < 10^{-10}$	$- < 10^{-10}$	-1.8×10^{-7}	-1.0×10^{-2}	0.58	0.49	
